# Niclosamide activates the NLRP3 inflammasome by intracellular acidification and mitochondrial inhibition

**DOI:** 10.1038/s42003-018-0244-y

**Published:** 2019-01-03

**Authors:** Uyen Thi Tran, Toshimori Kitami

**Affiliations:** YCI Laboratory for Cellular Bioenergetic Network, RIKEN Center for Integrative Medical Sciences, 1-7-22 Suehiro-cho, Tsurumi-ku, Yokohama, Kanagawa 230-0045 Japan

## Abstract

The NLRP3 inflammasome is unique among pattern recognition receptors in using changes in cellular physiology as a mechanism for sensing host danger. To dissect the physiological network controlling inflammasome activation, we screened for small-molecule activators and suppressors of IL-1β release in macrophages. Here we identified niclosamide, a mitochondrial uncoupler, as an activator of NLRP3 inflammasome. We find that niclosamide inhibits mitochondria and induces intracellular acidification, both of which are necessary for inflammasome activation. Intracellular acidification, by inhibiting glycolysis, works together with mitochondrial inhibition to induce intracellular ATP loss, which compromises intracellular potassium maintenance, a key event to NLRP3 inflammasome activation. A modest decline in intracellular ATP or pH within an optimal range induces maximum IL-1β release while their excessive decline suppresses IL-1β release. Our work illustrates how energy metabolism converges upon intracellular potassium to activate NLRP3 inflammasome and highlights a biphasic relationship between cellular physiology and IL-1β release.

## Abstract (web summary)

Uyen Thi Tran and Toshimori Kitami show that niclosamide activates the NLRP3 inflammasome through a modest intracellular acidification or ATP loss while an excessive decline of intracellular pH or ATP suppresses it. This study uncovers a biphasic immune response that accommodates cellular energy level.

## Introduction

The mammalian innate immune system detects a wide range of dangers to the host by using extracellular and intracellular pattern recognition receptors that bind pathogen specific molecules (pathogen-associated molecular patterns), as well as molecules released from damaged or dying cells (damage-associated molecular patterns). A subset of intracellular pattern recognition receptors form high-molecular-weight complexes called inflammasomes, which activates inflammatory caspases^1^. Among the inflammasomes^2^, nucleotide-binding oligomerization domain, leucine-rich repeat and pyrin domain containing (NLRP) 1, NLRP3, NLRP6, and pyrin appear to lack specific ligands but instead uses cellular homeostatic changes as a danger sensing mechanism^3^. The NLRP3 inflammasome is unique among these in that it is activated by variety of substances that accumulate during age-associated diseases. These include cholesterol crystals in atherosclerosis^4^, ceramide, fatty acids, and islet amyloid polypeptide in type 2 diabetes^5–7^, beta amyloid in Alzheimer’s disease^8^, uric acid crystal in gout^9^, as well as silica and asbestos present in environmental pollution^10^. *NLRP3* knockout mice are protected from these chronic inflammatory diseases^5,6,8,9,10^ and interleukin (IL)-1β antagonists are used clinically for treatment of gout and clinical trials are ongoing for type 2 diabetes^11^ and atherosclerotic disease^12^. New efforts are underway to identify small-molecule inhibitors of the NLRP3 inflammasome^13^.

Activation of the NLRP3 inflammasome is a two-step process^14^ in which signal 1 (also called ‘priming’), consisting of NF-κB activation, induces expression of NLRP3 and pro-IL-1β. This is followed by signal 2, a disruption in cellular physiology, which triggers the formation of an inflammasome protein complex consisting of NLRP3, the adaptor apoptosis-associated speck-like protein containing a caspase recruitment domain (ASC), and pro-caspase-1 (CASP1). This leads to autocleavage and activation of caspase-1 followed by the maturation and release of pro-inflammatory cytokines IL-1β or IL-18 via pyroptotic cell death. At least three physiological processes have been identified that can provide signal 2 for the NLRP3 inflammasome. These include potassium efflux^15,16^, elevation in mitochondrial reactive oxygen species (ROS)^17^, and lysosomal damage^18^. However, it remains unclear whether these physiological processes act independently on the NLRP3 inflammasome or whether they form an integrated network converging upon a common physiological output. In addition, the role of mitochondria varies widely between reports, from being a necessary and sufficient step^17^ to neither necessary nor sufficient^19^ for NLRP3 inflammasome activation. A key challenge in investigating the NLRP3 inflammasome pathway is to unravel the multiple cellular physiological changes that occur because of a single perturbation, and to understand how these physiological changes are quantitatively integrated into a single immune output.

Combinatorial screens have been applied successfully in the past to systematically dissect the inner wiring of complex systems as well as to reveal new properties that arise from such systems^20,21^. Here we performed chemical screens searching for new activators of the NLRP3 inflammasome and suppressors unique to each activator in human THP-1 macrophages and in mouse bone marrow-derived macrophages (BMDMs). From this data set, we identified niclosamide as an activator of the NLRP3 inflammasome. We find that niclosamide engages two targets, mitochondria and acidic organelles, both of which are necessary to induce NLRP3 inflammasome activation. Niclosamide transfers protons from acidic organelles to cytosol, leading to intracellular acidification and suppression of glycolysis. Glycolytic inhibition, combined with mitochondrial inhibitory action of niclosamide, decreases intracellular ATP level, leading to intracellular potassium loss, a key event to NLRP3 inflammasome activation. We also find that a modest decline in intracellular ATP or pH within an optimal range induces maximum IL-1β release while their excessive decline suppresses IL-1β release. Our study illustrates how energy metabolism converges upon intracellular potassium to activate NLRP3 inflammasome and highlights a biphasic relationship between physiological parameters and IL-1β release.

## Results

### Discovery of new NLRP3 inflammasome activator niclosamide

**Fig. 1:**
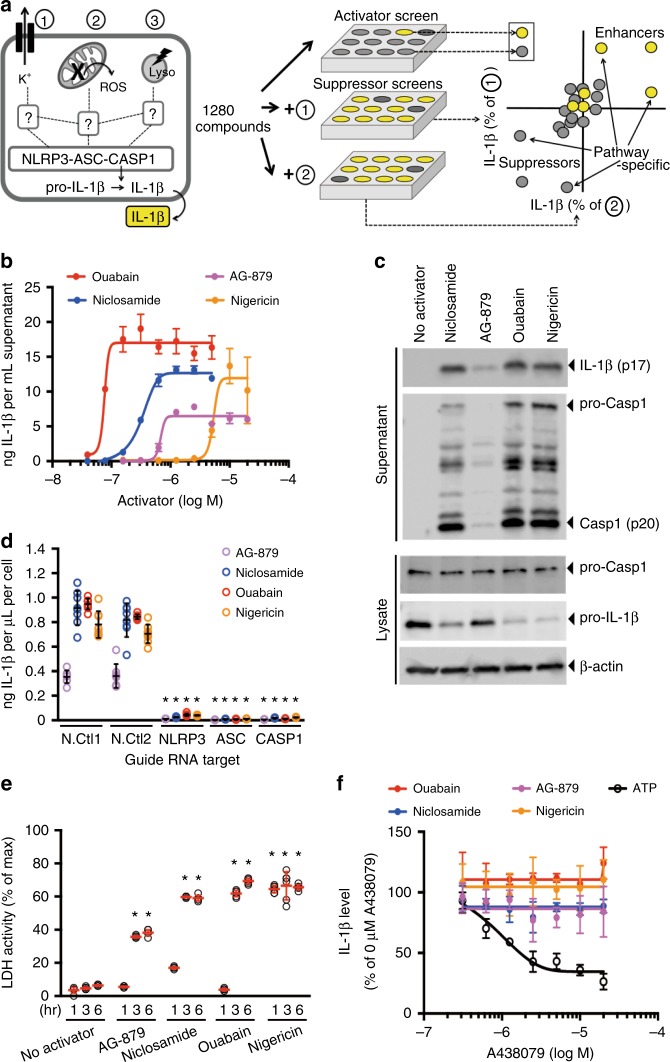
Identification of new NLRP3 inflammasome activators. **a** Schematic of activator-suppressor screens for IL-1β release. **b** Effect of the top 3 activators (6 h treatment, this notation is used throughout the figure legends) on IL-1β release (nigericin; positive control). **c** Immunoblot of biologically active IL-1β (p17) and caspase-1 (Casp1; p20) following activator treatment (3 h). Data represent two independent experiments; full gel images are in Supplementary Figure 14. **d** Effect of gene knockout of NLRP3 inflammasome components on IL-1β release (6 h). Guide RNA corresponding to non-targeting negative controls (N.Ctl) or gene-specific targets are indicated. **e** Effect of activators on LDH release (1 h, 3 h, 6 h). **f** Effect of P2RX7 antagonist on IL-1β release (1 h A438079 pretreatment followed by 6 h activator treatment in the presence of A438079). Data are mean ± s.d.; *n* = 3 (**b**, **f**), 6 (**e**), or 8 (**d**) biological replicates from one independent experiment; data are representative of two independent experiments. *P* values were determined by two-way ANOVA followed by Tukey’s multiple testing (**d**, **e**). *P < 0.0001 compared to N.Ctl1 (**d**) or no activator control of each time point (**e**). THP-1 macrophages (**b**, **c**, **e**, **f**) or THP-1 macrophages expressing guide RNA (**d**) were used for measurements. Activators were used at 1 μM (niclosamide), 2.5 μM (AG-879), 1 μM (ouabain), 20 μM (nigericin), or 5 mM (ATP) (**c**–**f**)

To systematically dissect pathways involved in NLRP3 inflammasome activation, we devised a screening strategy searching for small-molecule activators of the NLRP3 inflammasome, followed by identification of suppressors specific to each activator (Fig. 1a, Supplementary Fig. 1a–d). Using the THP-1 macrophage cell line and IL-1β release as a readout, we identified top 132 activators of IL-1β release out of 1280 compounds, corresponding to IL-1β release level greater than 30% of ouabain, an inhibitor of Na^+^-K^+^ ATPase and a previously identified activator of NLRP3 inflammasome^15^. Of these 132 activators, 60 reproducibly induced IL-1β release (>30% of ouabain) at 40 μM concentration and were inhibited (>90% suppression) in *NLRP3* and *CASP1* knockout cells (Supplementary Fig. 1a, Supplementary Table 1, Supplementary Data 1). Of these 60 validated activators, three of them induced robust IL-1β release at nanomolar concentrations (Fig. 1b). These were ouabain, a positive control, niclosamide, a mitochondrial uncoupler used as an anti-helminthic, and AG-879, a kinase library compound with unknown target. The levels of IL-1β release were close to nigericin, a potent inflammasome activator due to its ability to induce potassium efflux. We found that these activators induced caspase-1 activation and cleavage of pro-IL-1β (Fig. 1c) via NLRP3 inflammasome components (Fig. 1d), as assessed by analysis of CRISPR knockout cells. These activators also induced lactate dehydrogenase (LDH) release indicating induction of pyroptosis (Fig. 1e). IL-1β release was not inhibited by purinergic receptor P2X7 (P2RX7) antagonists, suggesting that NLRP3 inflammasome activation is not mediated by extracellular ATP released from dying cells (Fig. 1f). Compared to nigericin, these activators required longer incubation time for IL-1β release suggesting that their mechanism of action require multiple steps (Supplementary Fig. 2a). Analogous screen for activators in lipopolysaccharide (LPS) primed mouse bone marrow-derived macrophages (BMDMs) also identified 69 activators at 40 μM screening concentration, defined as IL-1β level greater than 10% of nigericin control (Supplementary Fig. 1b, Supplementary Table 2, Supplementary Data 2). We used nigericin as a reference because ouabain requires greater than 40 μM concentration to achieve IL-1β release in BMDM^15^. Of the 69 activators in BMDM, 23 of them overlapped with 60 validated activators in THP-1 macrophages. Of these 69 activators in BMDM, niclosamide was the only activator that induced IL-1β release at nanomolar concentration (Supplementary Fig. 3a, b). In BMDM, IL-1β release by niclosamide was inhibited by NLRP3 inflammasome inhibitor MCC950^22^ (Supplementary Fig. 3c) and niclosamide also induced IL-1β release in peritoneal and alveolar macrophages (Supplementary Fig. 3d and 3e). In BMDM, niclosamide also required longer incubation than nigericin to observe IL-1β release (Supplementary Fig. 3f). Thus, our screen identified niclosamide as an activator of the NLRP3 inflammasome, providing us with small-molecule tool in which to investigate the role of mitochondria in inflammasome activation.

### Global landscape of activator-suppressor interaction

**Fig. 2:**
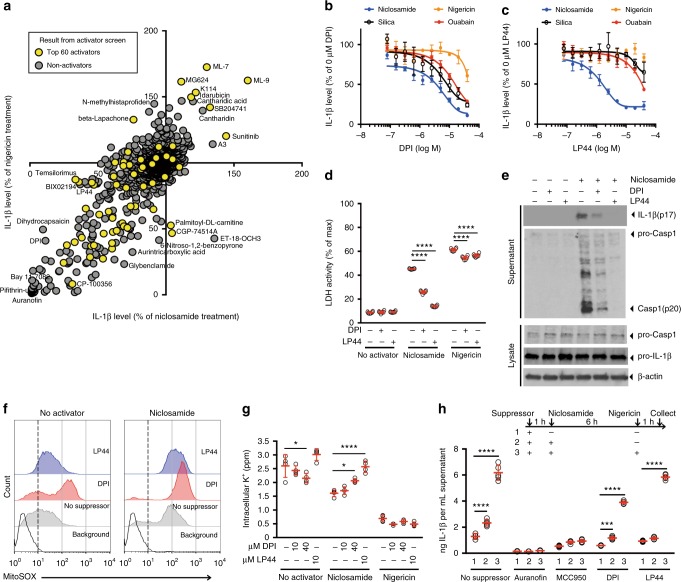
Niclosamide-biased suppressors prevent intracellular potassium loss. **a** Results of suppressor screens using nigericin (20 μM) or niclosamide (5 μM) as activators. Yellow circles indicate the top 60 validated activators. **b**, **c** Effect of niclosamide-biased suppressors on IL-1β release. **d** Effect of suppressors on LDH release. **e** Immunoblot of biologically active IL-1β (p17) and caspase-1 (Casp1; p20) after suppressor treatment. Data represent two independent experiments. Full gel images are shown in Supplementary Figure 14. **f** FACS analysis of mitochondrial ROS. Data represent three independent experiments. **g** Effect of suppressors on intracellular potassium level. **h** Rescue of IL-1β release by nigericin following suppressor treatment. Numbers (1–3) represent experimental scheme illustrated above the figure panel. Data are mean ± s.d.; *n* = 3 (**b**, **c**), 4 (**g**), 5 (**h**), or 6 (**d**) biological replicates from one independent experiment; data are representative of two independent experiments. *P* values were determined by two-way ANOVA followed by Tukey’s multiple testing. *P < 0.05, ***P < 0.001, ****P < 0.0001 as indicated. THP-1 macrophages (**a**–**e**, **h**) or THP-1 macrophages expressing *NLRP3* guide RNA (**f**, **g**) were used for measurements. 1 h suppressor treatment (10 μM DPI, 10 μM LP44, or as indicated) was followed by 1 h (**f**) or 6 h (**b**–**e**, **g**–**h**) activator treatment (1 μM niclosamide, 20 μM nigericin, 1 μM ouabain, or 250 μg mL^−1^ silica) in the presence of suppressors (**b**–**h**). Nigericin rescue (**h**) (20 μM) for 1 h was performed following 6 h niclosamide treatment

To identify molecular components involved in mitochondria-mediated NLRP3 inflammasome activation, we searched for suppressors of niclosamide-induced IL-1β release in THP-1 macrophages. To assess whether the identified suppressors act specifically upon niclosamide or act more generally across a variety of activators, we performed a parallel suppressor screen using nigericin, a potassium ionophore, as an activator (Supplementary Data 1, Supplementary Table 3). We found that many compounds showed enhancer or suppressor effects at equal magnitude in both niclosamide and nigericin treated cultures, suggesting that the molecular components involved in NLRP3 activation by mitochondrial and potassium pathways are largely shared (Fig. 2a). Interestingly, when we mapped the top 60 validated activators to the enhancer/suppressor profiles (Fig. 2a; yellow circles), we found that some of the suppressors were also activators, suggesting that certain combinations of activators act antagonistically on IL-1β release. This suggests that maximum activation of the NLRP3 inflammasome and IL-1β release involve optimized combinations of cellular perturbations rather than a random addition of cytotoxic events.

### Niclosamide suppressors do not inhibit mitochondrial ROS

To cleanly dissect the molecular pathways involved in mitochondria-mediated NLRP3 inflammasome activation, we selected the top suppressors of niclosamide-induced IL-1β release (Supplementary Fig. 1c, Supplementary Table 3, Supplementary Data 1). We then filtered out suppressors that were primary hits in the activator screen (top 132 activators) to avoid suppression resulting from antagonism between two activators. We tested these top 80 “clean” suppressors at lower doses, four of which suppressed niclosamide-induced IL-1β release at low micromolar to nanomolar concentration. These compounds did not induce cell toxicity as assessed by calcein assay (Supplementary Data 1) and they showed selectivity for NLRP3 but not to absent in melanoma 2 (AIM2) inflammasome activator (Supplementary Fig. 4a). Among these four suppressors, diphenyleneiodonium (DPI) and LP44 showed niclosamide-biased suppression in THP-1 macrophage cells (Fig. 2b, c) suggesting that they may provide clues to the mechanism of action of niclosamide. Inhibition of IL-1β release can be achieved as a pre-treatment (starting 1 h prior to niclosamide addition), co-treatment, as well as 1 h post-niclosamide treatment (Supplementary Fig. 4b). Combination of DPI and LP44 did not deviate substantially from the Bliss independence model (Supplementary Fig. 4c). DPI and LP44 also blocked niclosamide-induced LDH release (Fig. 2d) and caspase-1 activation (Fig. 2e). DPI is a known inhibitor of NADPH oxidase^23^ and LP44 is a 5-hydroxytryptamine (serotonin) receptor 7 (5-HT_7_) agonist^24^. However, additional agonists of 5-HT_7_ did not inhibit niclosamide-induced IL-1β release (Supplementary Fig. 4d), suggesting that other targets are involved in the action of LP44. In addition, pre-treatment with DPI and LP44 alone elevated rather than suppressed mitochondrial ROS, and this effect was enhanced upon niclosamide treatment in *NLRP3* knockout THP-1 macrophage cells (Fig. 2f). We used *NLRP3* knockout THP-1 macrophages to ensure that elevation in mitochondrial ROS is not the result of NLRP3 inflammasome activation but rather an effect of niclosamide upstream of NLRP3. Elevation in mitochondrial ROS suggests that DPI is suppressing IL-1β release via mechanism other than NADPH oxidase inhibition as we would expect suppression of ROS. We also found that DPI and LP44 suppressed H_2_O_2_-induced IL-1β release in THP-1 macrophages without inhibiting mitochondrial ROS production (Supplementary Fig. 4e and 4f) suggesting that suppression of mitochondrial ROS is not the mechanism of DPI and LP44. In BMDM, DPI and LP44 also showed niclosamide-biased suppression (Supplementary Fig. 5a, b) and elevation in mitochondrial ROS (Supplementary Fig. 5c).

To more directly examine the role of mitochondrial ROS in niclosamide-induced NLRP3 inflammasome activation, we also used a mitochondria-targeted antioxidant mitoTEMPO. However, mitoTEMPO requires mitochondrial membrane potential for import and retention in mitochondria, which is dissipated by the action of niclosamide. Therefore, we did not observe any decrease in mitochondrial ROS (Supplementary Fig. 6a). Instead, we observed an increase in mitochondrial ROS at higher mitoTEMPO concentrations, accompanied by a decrease in niclosamide-induced IL-1β release (Supplementary Fig. 6b). We also examined whether oxidized mitochondrial DNA^25^ is involved in niclosamide-induced NLRP3 inflammasome activation by decreasing mitochondrial DNA levels with a 4-week ethidium bromide treatment. We found that ethidium bromide treatment at a sublethal dose resulted in a 50% reduction in mitochondrial DNA copy number (Supplementary Fig. 6c) accompanied by a decrease in both niclosamide- and nigericin-induced IL-1β release (Supplementary Fig. 6d). Interestingly, however, ethidium bromide treatment also decreased IL-1β mRNA levels (Supplementary Fig. 6e), suggesting that decreased priming (signal 1) may be partly responsible for reduced IL-1β release. Overall, our results suggest that mechanisms outside of mitochondrial ROS are responsible for niclosamide-biased suppression of IL-1β release by DPI and LP44.

### Niclosamide suppressors prevent intracellular potassium loss

Among the several pathways leading to NLRP3 inflammasome activation, a recent study has pointed to potassium efflux as a terminal step to NLRP3 activation^15^. Therefore, we tested whether niclosamide, along with its suppressors, alter intracellular potassium levels. Niclosamide treatment decreased intracellular potassium level in *NLRP3* knockout THP-1 macrophages, and this decrease was prevented by pretreatment with DPI and LP44 (Fig. 2g). The suppression of IL-1β release by DPI and LP44 can be rescued with potassium ionophore nigericin (Fig. 2h), supporting that DPI and LP44 suppress NLRP3 inflammasome activation by inhibiting intracellular potassium loss.

In addition to THP-1 macrophages, we also performed suppressor screen for niclosamide in BMDM (Supplementary Table 4, Supplementary Data 2). Among the top three niclosamide-biased suppressors (Supplementary Fig. 1d), we identified potassium channel (*K*_v_1.3 and *K*_v_1.5) inhibitor Psora-4. In addition, we identified potassium channel *K*_v_1.5 inhibitor mephetyl tetrazole among the top 36 niclosamide-biased suppressors. These niclosamide-biased suppressors, along with LP44, showed selectivity for NLRP3 but not to AIM2 inflammasome activator (Supplementary Fig. 7a). Importantly, suppression of niclosamide-induced IL-1β release can be rescued with nigericin post-treatment (Supplementary Fig. 7b), suggesting that niclosamide-biased suppressors prevent intracellular potassium loss in THP-1 macrophages and in BMDM.

### Niclosamide engages two targets for robust IL-1β release

**Fig. 3:**
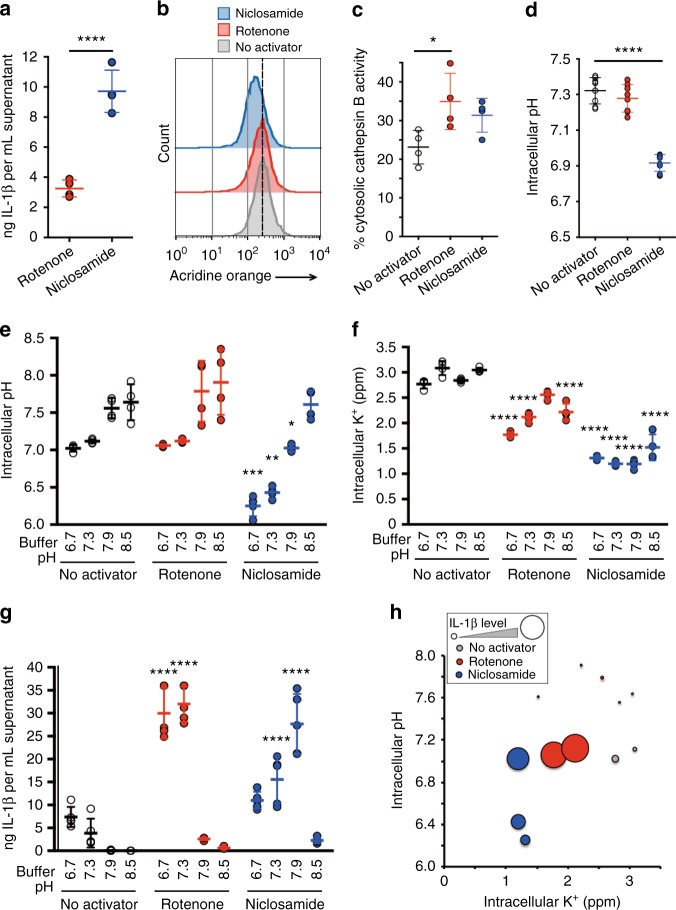
Niclosamide induces intracellular acidification. **a** Effect of mitochondrial inhibitors (6 h) on IL-1β release. **b** FACS analysis of proton levels in acidic compartments treated with mitochondrial inhibitors (1 h). Data represent two independent experiments. **c** Effect of mitochondrial inhibitors (1 h) on lysosomal membrane permeabilization (LMP). **d** Effect of mitochondrial inhibitors (1 h) on intracellular pH. **e** Effect of mitochondrial inhibitors (1 h) in different pH buffers on intracellular pH. **f** Effect of mitochondrial inhibitors (6 h) in different pH buffers on intracellular potassium levels. **g** Effect of mitochondrial inhibitors (6 h) in different pH buffers on IL-1β release. **h** Relationship between intracellular pH, potassium, and IL-1β release using data from **e** to **g**. The size of the circle is in proportion to the level of IL-1β release. Data are mean ± s.d.; *n* = 4 (**a**, **c**, **e**, **f**), 5 (**g**), or 8 (**d**) biological replicates from one independent experiment; data are representative of two independent experiments. *P* values were determined by a two-tailed Student’s *t* test (**a**) or one-way (**c**, **d**) and two-way (**e**–**g**) ANOVA followed by Tukey’s multiple testing. *P < 0.05, **P < 0.01, ***P < 0.001, ****P < 0.0001 compared to no activator control for each pH buffer type or as indicated. THP-1 macrophages expressing negative control (N.Ctl1) guide RNA (**a**, **g**) or *NLRP3* guide RNA (**b**–**f**) were used for measurements. Mitochondrial inhibitors were used at 5 μM (rotenone) or 1 μM (niclosamide) (**a**–**g**)

To test whether other inhibitors of mitochondria, aside from niclosamide, can robustly activate NLRP3 inflammasome, we performed a focused screen of 23 mitochondrial inhibitors identified from prior screens^26,27^ as well as gold standard inhibitors of mitochondrial electron transport chain. Surprisingly, niclosamide remained the only mitochondrial inhibitor with high IL-1β release level (Supplementary Fig. 8a) suggesting that niclosamide may engage additional targets outside of mitochondria for robust IL-1β release. A previous study showed that, aside from acting as a mitochondrial uncoupler by dissipating the proton gradient of mitochondria, niclosamide also dissipates the proton gradient of acidic cellular compartments such as late endosomes and lysosomes^28^. Given that these acidic organelles are abundant in macrophages, we hypothesized that NLRP3 inflammasome activation by niclosamide also involves transfer of protons from acidic organelles to the cytosol. To test this hypothesis, we compared the effects of niclosamide against rotenone, a complex I inhibitor of the electron transport chain in mitochondria. We expect rotenone, a direct inhibitor of complex I, to not have any proton transfer capability and therefore do not affect the pH gradient of cytosol (Supplementary Fig. 9a). In contrast, niclosamide, by having a proton transfer capability, may also transfer protons from acidic organelles to cytosol, causing intracellular acidification. For IL-1β measurement, we used control cells whereas for physiological measurements we used *NLRP3* knockout cells. We found that niclosamide treatment resulted in a 3-fold higher level of IL-1β compared to rotenone (Fig. 3a). As expected, niclosamide, but not rotenone treatment, decreased protons in acidic organelles (Fig. 3b). This was not due to a higher level of lysosomal damage by niclosamide treatment (Fig. 3c). Niclosamide also induced intracellular acidification (Fig. 3d), suggesting that protons from acidic organelles were transferred to the cytosolic space.

We next asked whether phenocopying the dual action of niclosamide, mitochondrial inhibition and intracellular acidification, leads to intracellular potassium loss and an activation of IL-1β release. We artificially lowered intracellular pH by lowering the pH of the extracellular buffer (Fig. 3e). A decrease in intracellular pH alone did not induce a decrease in intracellular potassium level (Fig. 3f). However, the combination of intracellular acidification with rotenone treatment led to a robust decrease in intracellular potassium level (Fig. 3f) and an increase in IL-1β release (Fig. 3g), similar to the level achieved by niclosamide. In the case of niclosamide, because it triggers intracellular acidification, niclosamide decreased intracellular pH and potassium level in pH 7.9 buffer accompanied by robust IL-1β release (Fig. 3e-g). However, lower pH buffers (pH 6.7 and 7.3) led to an additional decline in intracellular pH (Fig. 3e), accompanied by a decline in IL-1β release despite loss of intracellular potassium (Fig. 3f–h). We were unable to detect the combinatorial effect of rotenone and acidic buffer in BMDM, although IL-1β release by niclosamide was inhibited upon treatment with lower pH buffer, similar to the THP-1 macrophages (Supplementary Fig. 9b and 9c). In addition, we observed combinatorial effect of acidic buffer and rotenone in human CD14^+^ monocyte derived macrophages (Supplementary Fig. 9d). These observations suggest that while combining intracellular acidification with mitochondrial inhibition induces intracellular potassium loss and activates IL-1β release, a severe intracellular acidification suppresses IL-1β release.

### Two-hit model of NLRP3 activation involving mitochondria

**Fig. 4:**
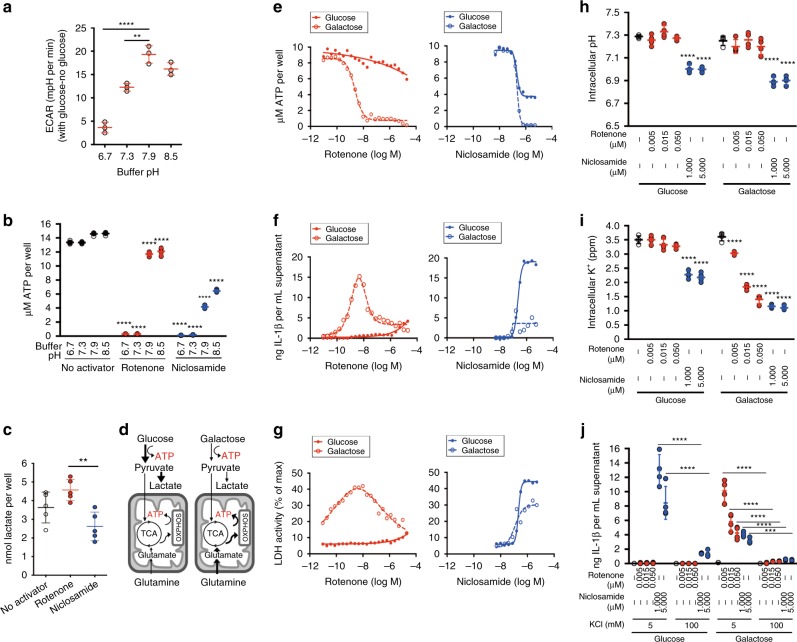
Two-hit inhibition of ATP production leads to robust IL-1β release. **a** Effect of buffer pH on glycolysis rate as measured by extracellular acidification rate (ECAR) with 10 mM glucose, subtracting basal ECAR value without glucose. **b** Effect of mitochondrial inhibitors (6 h) in different pH buffers on intracellular ATP levels. **c** Effect of mitochondrial inhibitors (1 h) on intracellular lactate levels. **d** Schematics illustrating fuel utilization under two media conditions, glucose and galactose. **e**–**g** Effect of mitochondrial inhibitors (6 h) on (**e**) intracellular ATP levels, (**f**) IL-1β release, and (**g**) LDH release in glucose- or galactose-containing media. Each dot corresponds to a single experimental replicate; data are representative of two independent experiments. **h** Effect of mitochondrial inhibitors (1 h) on intracellular pH level in glucose- or galactose-containing media. **i** Effect of mitochondrial inhibitors (6 h) on intracellular potassium level in glucose- or galactose-containing media. **j** Effect of high (100 mM) versus normal (5 mM) extracellular potassium in suppressing IL-1β release. Data are mean ± s.d.; *n* = 3 (**a**), *n* = 4 (**h**–**j**) or 5 (**b**, **c**) biological replicates from one independent experiment; data are representative of two independent experiments. *P* values were determined by one-way (**a**, **c**) and two-way (**b**, **h**–**j**) ANOVA followed by Tukey’s multiple testing. *P < 0.05, **P < 0.01, ***P < 0.001, ****P < 0.0001 compared to the no activator control for each pH buffer or media type (**b**, **h**, **i**) or as indicated (**a**, **c**, **j**). THP-1 macrophages expressing negative control (N.Ctl1) guide RNA (**f**) or *NLRP3* guide RNA (**b**, **c**, **e**, **g**–**i**) or without vector expression (**a**, **j**) were used for measurements. Mitochondrial inhibitors were used at 5 μM (rotenone) or 1 μM (niclosamide) (**b**, **c**) or as indicated

To understand the link between mitochondrial inhibition and intracellular acidification, we searched in the literature for pH-sensitive process that shares common output as mitochondria. We considered whether glycolysis, which is suppressed at acidic pH^29^, interacts with mitochondria as both pathways produce ATP. We first measured the effect of buffer pH on the rate of glycolysis. We found that glycolysis rate, as measured by extracellular acidification rate after the addition of glucose, were slowest in low pH buffers (Fig. 4a). Next, we considered whether acidic pH, which inhibits glycolysis, in combination with mitochondrial inhibition results in loss of intracellular ATP as both glycolytic and mitochondrial ATP synthesis are inhibited. We found that the combination of low pH buffers with rotenone treatment potently decreased intracellular ATP levels (Fig. 4b). Niclosamide decreased intracellular ATP level in more basic pH buffers as niclosamide itself induces intracellular acidification. Niclosamide also decreased intracellular lactate levels, an output of glycolysis, while rotenone elevated them (Fig. 4c), suggesting that niclosamide, but not rotenone, inhibits glycolysis.

To more directly assess the impact of ATP synthesis upon IL-1β release, we changed the carbon source in the culture media from glucose to galactose, which bypasses glycolysis and engages only mitochondria for ATP production (Fig. 4d). Galactose media induced a minor decrease in *IL-1*β mRNA, suggesting that the priming step was largely unaffected (Supplementary Fig. 10a). With glucose media, rotenone decreased intracellular ATP only by 33% (Fig. 4e) as glycolysis can compensate for inhibition of mitochondrial ATP production. Rotenone treatment in glucose media also modestly increased IL-1β (Fig. 4f, Supplementary Fig. 10b) and LDH (Fig. 4g) levels. With galactose media, rotenone decreased intracellular ATP by 85% (Fig. 4e), as glycolysis cannot compensate for inhibition of mitochondrial ATP production. This ATP decrease accompanied large elevation in IL-1β (Fig. 4f) and LDH (Fig. 4g) release. At higher concentrations, rotenone depleted intracellular ATP and decreased IL-1β release. By contrast, niclosamide decreased intracellular ATP by 60% even in glucose media (Fig. 4e) as glycolysis is inhibited by intracellular acidification, accompanied by increased IL-1β (Fig. 4f, Supplementary Fig. 10b) and LDH release (Fig. 4g). In galactose media, niclosamide depleted intracellular ATP (Fig. 4e) and decreased IL-1β release (Fig. 4f). We also found similar results for mitochondrial inhibitors in glucose and galactose media in BMDM cultures (Supplementary Fig. 10c and 10d). In addition, 2-deoxyglucose, a glycolysis inhibitor, in combination with rotenone achieved similar results as galactose media in THP-1 macrophages (Supplementary Fig. 10e–g).

We next measured intracellular pH under two carbon sources to ensure that robust IL-1β release by rotenone did not result from intracellular pH changes. The intracellular pH (Fig. 4h) following rotenone treatment under glucose or galactose remained constant suggesting that inhibition of ATP synthesis was responsible for NLRP3 inflammasome activation. Next, we tested whether a decrease in intracellular ATP led to a loss of intracellular potassium as potassium is maintained by Na^+^–K^+^ ATPase, one of the most ATP consuming processes in the cell^30^. Rotenone treatment in galactose media or niclosamide treatment in glucose media strongly decreased intracellular potassium level (Fig. 4i). We found that IL-1β release is inhibited with high extracellular potassium (Fig. 4j), which prevents potassium efflux, suggesting that intracellular potassium loss was responsible for IL-1β release following two-hit inhibition of ATP synthesis. We also observed a robust intracellular potassium loss for niclosamide treatment in galactose media (Fig. 4i), suggesting that a severe decrease in intracellular ATP, despite loss of intracellular potassium, becomes inhibitory for NLRP3 inflammasome activation. Overall, our results suggest that intracellular acidification, by suppressing glycolysis, works with mitochondrial inhibition to decrease intracellular ATP, thereby compromising intracellular potassium maintenance and activating IL-1β release. However, a severe decline in intracellular ATP inhibits IL-1β release, similar to the biphasic effect of pH upon IL-1β release.

### AG-879 is also a mitochondrial uncoupler

**Fig. 5:**
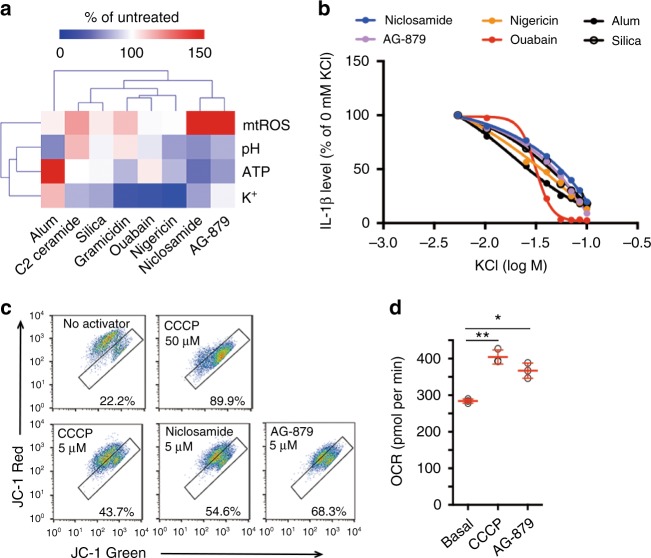
Global profiling of cell physiology. **a** Heat map of physiological parameters across a panel of activators. Individual data are shown in Supplementary Figure 11. **b** Effect of extracellular potassium levels on IL-1β release (1 h high potassium pretreatment followed by 6 h activator treatment in the presence of high potassium). Each dot corresponds to a single experimental replicate; data are representative of two independent experiments. **c** FACS analysis of mitochondrial membrane potential after 15 min treatment with DMSO (No activator), CCCP, niclosamide, or AG-879. **d** Changes in oxygen consumption rate (OCR) before (basal) and after treatment with CCCP (1 μM) or AG-879 (1 μM). Data are mean ± s.d.; *n* = 3 biological replicates from one independent experiment; data are representative of two independent experiments. *P* values were determined by one-way ANOVA followed by Tukey’s multiple testing. *P < 0.05, **P < 0.01. THP-1 macrophages expressing *NLRP3* guide RNA (**a**) or normal THP-1 macrophages (**b**–**d**) were used for measurements. Activators were used at 5 μM (rotenone), 1 μM (niclosamide), 2.5 μM (AG-879), 1 μM (ouabain), 20 μM (nigericin), 10 μM (gramicidin), 125 μM (C2 ceramide), 500 μg mL^−1^ (alum), or 250 μg mL^−1^ (silica) (**a**, **b**)

Our systematic analysis of niclosamide function pointed to a handful of critical cellular processes controlling the activation of the NLRP3 inflammasome. To investigate whether other activators also alter these key cellular processes, we performed global profiling of physiological parameters across a variety of activators (Fig. 5a, Supplementary Fig. 11a–11d). We found that some of the robust activators, such as nigericin, can alter more than one key process, including intracellular ATP, pH, and potassium levels, while others, such as ouabain, are limited to effects on potassium. Interestingly, of the four processes we examined, loss of intracellular potassium was the common mechanism shared by most of the activators tested, supporting the concept that potassium efflux is the common and terminal step leading to NLRP3 inflammasome activation. In addition, high extracellular potassium inhibited IL-1β release by these activators (Fig. 5b), including AG-879, the other potent activator identified in our screen.

Global profiling of physiological parameters also revealed that AG-879, a newly identified activator of the NLRP3 inflammasome, had a strikingly similar profile to niclosamide, suggesting a similar mechanism of action. Consistent with our hypothesis, AG-879 decreased mitochondrial membrane potential, similar to niclosamide and the positive control CCCP (Fig. 5c). We also measured the oxygen consumption rate (OCR), which is elevated during mitochondrial uncoupling but not during cell toxicity, to eliminate the possibility of any cell toxicity-induced drop in membrane potential. We found that AG-879 elevated OCR (Fig. 5d), suggesting that it is a mitochondrial uncoupler whose effect on NLRP3 inflammasome activation may involve its uncoupling activity rather than any kinase targeting activity. Therefore, some of the mitochondrial uncouplers are likely to possess NLRP3 inflammasome activating activity.

### Biphasic control of IL-1β release by cellular physiology

**Fig. 6:**
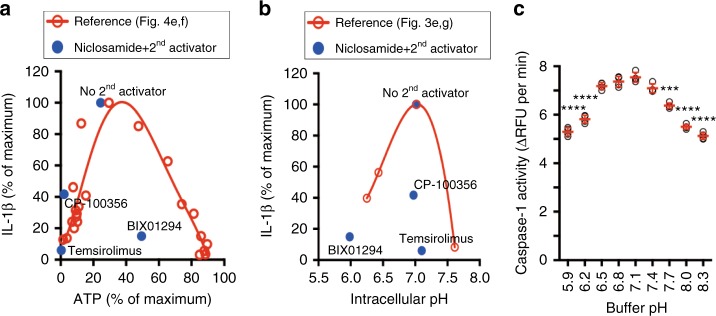
Biphasic relationship between IL-1β release and physiological parameters. **a** Relationship between intracellular ATP levels and IL-1β release. The reference curve was drawn using data from Fig. 4e, f. **b** Relationship between intracellular pH and IL-1β release The reference curve was drawn using data from Fig. 3e, g. Blue dots (**a**, **b**) indicate an antagonistic interaction between niclosamide and a second activator in Supplementary Fig. 11e-g. **c** pH dependency of caspase-1 activity in THP-1 macrophage cell lysates. Data are mean ± s.d.; *n* = 4 technical replicates from one independent experiment; data are representative of two independent experiments. *P* values were determined by one-way ANOVA followed by Tukey’s multiple testing. ***P < 0.001, ****P < 0.0001 compared to pH 7.4 buffer

Our analysis of niclosamide function revealed that a decline in intracellular ATP and pH had a biphasic effect on IL-1β release, such that a modest decrease activates, but a severe decrease inhibits IL-1β release. We therefore hypothesized that if two activators decrease ATP or pH, they would activate IL-1β release when used alone but inhibit IL-1β release when treated in combination due to the severe decline in ATP or pH. Our screen for activators of IL-1β release revealed 60 compounds that induced IL-1β release (Fig. 2a; yellow circles) but a subset, when combined with niclosamide or nigericin, inhibited rather than enhanced IL-1β release. We analyzed the antagonistic interactions between two activators (Supplementary Fig. 11e) and examined their effects on intracellular ATP (Supplementary Fig. 11f) and pH level (Supplementary Fig. 11g) in *NLRP3* knockout cells. Some of the antagonistic interactions between two activators resulted in a significant decline in intracellular ATP (Fig. 6a) and, in one instance, a severe decline in intracellular pH (Fig. 6b), suggesting that two activators targeting the same physiological pathway may push the physiological parameters outside of the optimal range for NLRP3 inflammasome activation. Similarly, some of the antagonistic interaction between activators from our screens in BMDM resulted in a significant decline in intracellular ATP level (Supplementary Fig. 12a–d).

A biphasic relationship between ATP/pH and IL-1β release may be explained by ATP- or pH-dependency of NLRP3 inflammasome activity or by the presence of at least two cellular processes that depend on ATP or pH and influence the NLRP3 inflammasome activation pathway in an opposing manner. We found that, analogous to the pH dependency of cytochrome c-mediated caspase activation^31^, caspase-1 activity is also pH dependent, with optimal activity at a slightly acidic pH (Fig. 6c). This suggests that the pH dependency of caspase-1 activity, in addition to inhibition of glycolysis, may be partly responsible for the biphasic effect of pH on IL-1β release.

## Discussion

We performed small-molecule screens to dissect the cellular physiology network regulating NLRP3 inflammasome activation. Through the identification of a NLRP3 inflammasome activator niclosamide, and the analysis of niclosamide-biased suppressors DPI and LP44, we found that elevation of mitochondrial ROS alone is insufficient for NLRP3 inflammasome activation. Instead, loss of mitochondrial ATP production, in combination with a blockade of glycolytic ATP production, determines the magnitude of IL-1β release through loss of intracellular potassium.

Our data are consistent with previous reports suggesting that mitochondrial inhibition alone does not drive robust activation of the NLRP3 inflammasome^19^, and that potassium efflux is the common pathway leading to activation^15^. On the other hand, other reports suggest a role for mitochondrial ROS^17^ or oxidized mitochondrial DNA^25^ in activating the NLRP3 inflammasome. We were unable to block an elevation in mitochondrial ROS using a mitochondria-specific antioxidant mitoTEMPO and we did observe a decrease in IL-1β release in mitochondrial DNA ‘reduced’ THP-1 macrophages that had been treated with ethidium bromide. Therefore, we cannot exclude a contribution of mitochondrial ROS and oxidized mitochondrial DNA to NLRP3 inflammasome activation. Instead, we highlight that multiple aspects of mitochondrial physiology can engage pathways leading to NLRP3 inflammasome activation and that intracellular ATP availability, rather than mitochondrial ROS, appears to be more predictive of the magnitude of IL-1β release.

Our two-hit model of NLRP3 inflammasome activation, which involves inhibition of both glycolytic and mitochondrial ATP production, suggests that disruption of one of these components, such as by rotenone treatment, does not induce robust NLRP3 inflammasome activation. Instead, when both pathways are engaged, we observed a robust activation response. This two-hit mechanism may help safeguard cells from constant fluctuations in cellular metabolism and only engage NLRP3 inflammasome activation in the presence of true danger signal when both ATP synthesis pathways are compromised. In addition, given that targets of caspase-1 cleavage include glycolysis and mitochondrial proteins^32,33^, caspase-1 activation itself may also trigger two-hit inhibition of ATP production, which may help enhance or secure commitment to NLRP3 inflammasome activation.

Importantly, two-hit inhibition of glycolytic and mitochondrial ATP production did not guarantee robust activation of the NLRP3 inflammasome. Indeed, an excessive decrease in ATP levels or pH prevented activation by pushing the physiological parameters outside of the optimal range for activation. This may be because each physiological parameter, such as intracellular ATP and pH, affects multiple cellular processes critical for NLRP3 inflammasome activation. For example, loss of intracellular ATP leads to activation via loss of intracellular potassium, but ATP binding to NLRP3 protein is required for inflammasome activation^34^, which may explain the biphasic effect of ATP on IL-1β release. In addition, we found that caspase-1 activity is pH dependent, analogous to the activity of other caspases^31^, which may be partly responsible for the biphasic effect of pH on IL-1β release.

The biphasic effects of ATP and pH on IL-1β release suggest that these physiological parameters are tuned to detect a specified range of cellular metabolic disruption^3^, without being reactive to small fluctuations in cellular physiology or to extreme cellular distress unrelated to pathogen invasion. Therefore, these optimal ranges may provide specificity as pattern recognition receptor without detecting a specific molecule. The optimal range and regulation of these physiological parameters may differ between cell types. We found that there was no synergism between acidic buffer and rotenone treatment in BMDM, although we did observe synergism between galactose media and rotenone treatment. The biphasic effects of pH and ATP on IL-1β release were investigated only in macrophages. Given that the NLRP3 inflammasome is active in cells other than macrophages, including neutrophils^35,36^, dendritic cells^37,38^, and keratinocytes^39^, further studies are warranted to understand the regulation of these physiological parameters and their optimal range for NLRP3 inflammasome activation across cell types and organisms.

It is important to note that not all antagonisms between activators result from excessive decrease in intracellular pH or ATP as described by the biphasic effects. Some of the antagonisms may result from off-target effect (or secondary target) of the first activator acting upon the second activator independently of pH or ATP. Another important point is that effects of activators upon pH or ATP may not be through the primary targets of compounds described in the databases but may be through additional targets not described in the literature. However, if two activators both affect intracellular ATP or pH, whether through off-target or primary target, they may induce excessive decrease in physiological parameters outside of optimal range for NLRP3 inflammasome activation, resulting in antagonistic interactions.

Our study focused on the measurement of IL-1β release in a population of cells, or cells within a well, treated with activators and suppressors. Although the output of NLRP3 inflammasome activation on a single cell level is binary, either presence or absence of pyroptotic cell death, not every cell undergo pyroptotic cell death upon addition of activators. Therefore, the amount of IL-1β release reflects the likelihood of NLRP3 inflammasome activation as intracellular physiology and metabolism are under slightly different condition among population of cells. A use of single-cell technologies to monitor caspase-1 activation and IL-1β release^40^ combined with measurement of intracellular physiological parameters^41,42^ will enable us to understand the cell-to-cell variability in NLRP3 inflammasome activation.

Although our chemical screens focused on signal 2 of the NLRP3 inflammasome, cellular metabolism also play an important role during signal 1 of the inflammasome activation. For example, previous study has shown that inhibition of glycolysis leads to suppression of pro-IL-1β transcription through suppression of HIF-1α^43^. Given that mitochondrial uncouplers has been shown to suppress IL-1β release when added during signal 1^44^ versus enhancement of IL-1β release during signal 2^45^, the same chemical may have opposing effect depending on which signal step is under investigation. Future screens involving signal 1 of the NLRP3 inflammasome may provide insights into similarities and differences in cellular processes regulating signal 1 and 2 of the NLRP3 inflammasome.

Our chemical screens revealed that hits in an NLRP3 inflammasome suppressor screen using IL-1β release may also include inflammasome activators because of antagonism between two activators. This finding highlights that future phenotypic screens for NLRP3 inflammasome inhibitors may require a counter-screen for activation of the NLRP3 inflammasome and measurement of key physiological processes. This would help determine whether the suppression results from alterations in cell physiological parameters critical for NLRP3 inflammasome activation. While we may avoid those suppressors and focus on potentially more direct inhibitors of NLRP3 inflammasome, these compounds may offer clues as to how physiological pathways upstream of NLRP3 inflammasome are wired together. In addition, screens designed to identify direct inhibitors of the NLRP3 inflammasome^13,46^ may also help minimize discovery of hit compounds that alter cell physiological processes^47^.

While our analysis focused on the mechanism of action of niclosamide and niclosamide-biased suppressors, DPI and LP44, our dataset (Supplementary Data 1, 2) contains other compounds capable of activating IL-1β release as well as suppressors that work across multiple activators. We found some suppressors of IL-1β release that were previously reported in the literature, including the anti-rheumatic compound auranofin^48^ and the anti-diabetic compound glyburide^49^, suggesting that other activators and suppressors in our dataset may serve as a potential resource for dissecting the mechanism of NLRP3 inflammasome activation. These small-molecule tools, used in a combinatorial fashion, can help unravel how multiple physiological pathways converge to regulate the decision switch and amplification of NLRP3 inflammasome activation.

## Methods

### Chemicals

The following chemicals were purchased from Sigma; LOPAC 1280 library (LO4200), niclosamide (N3510), AG-879 (T2067), nigericin (N7143), gramicidin (G5002), ouabain (O3125), C2 ceramide (A7191), ATP (A6419), DPI (D2926), LP44 (L9793), Psora-4 (P9872), mephetyl tetrazole (M7945), auranofin (A6733), rotenone (R8875), CCCP (C2759), mitoTEMPO (SML0737), aminopterin (A3411), berberine (B3251), bisacodyl (B1390), carvedilol (C3993), chlorprothixene (C1671), clemastine fumarate (SML0445), clofilium tosylate (C2365), clofoctol (C2290), clomiphene citrate (C6272), fluvastatin (SML0038), mefloquine (M2319), naftifine (N1790), nifuroxazide (46494), papaverine (P3510), pentamidine isethionate (P0547), phenformin (P7045), pimozide (P1793), sertraline (S6319), thiostrepton (T8902), vinpocetine (V6383), glucose (G7021), galactose (G0750), DMSO (D2650). Meclizine (sc-211779) and sertaconazole (c-280082) were purchased from Santa Cruz Biotechnology. Alum crystal (tlrl-alk), silica (tlrl-sio), and poly(dA:dT)/LyoVec (tlrl-patc) were purchased from InvivoGen. MCC950 (AG-CR1-3615-M005) was purchased from AdipoGen. A-438079 was purchased from Wako (012-25221). Activators and suppressors were prepared in DMSO between 10 mM and to 40 mM stock concentrations except for nigericin (5 mM in ethanol), gramicidin (10 mM in ethanol), alum (20 mg mL^−1^ in water), silica (5 mg mL^−1^ in water), and poly(dA:dT)/LyoVec (50 μg mL^−1^ in water).

### Cell culturing and isolation

THP-1 monocytes were purchased from ATCC (TIB-202) and grown in “THP-1 media” consisting of RPMI 1640 (ATCC modification; Thermo, A1049101) supplemented with 10% defined FBS (HyClone, SH30070.03), 1x penicillin/streptomycin (Thermo, 15140-122), and 0.05 mM beta mercaptoethanol (Sigma, M3148). Bone marrow-derived macrophages (BMDM) were cultured from bone marrow of 6 to 10-week-old C57BL/6 mice. Bone marrow cell suspensions from femurs and tibias of C57BL/6 mice were grown for 7 days in non-treated sterile 10 cm plates in “BMDM media” consisting of DMEM/F-12 (Thermo, 10565-018) supplemented with 10% heat-inactivated defined FBS, 1× penicillin/streptomycin, 1× non-essential amino acids (Thermo, 11140-050), and 20 ng mL^−1^ mouse M-CSF (BioLegend, 576406). Mouse peritoneal and alveolar macrophages were isolated from 6- to 10-week-old C57BL/6 mice^50^. Human CD14^+^ monocytes were purchased from Lonza and grown in RPMI (Sigma, R8758) supplemented with 10% heat-inactivated defined FBS, 1x penicillin/streptomycin and 50 ng mL^−1^ human recombinant M-CSF (Biolegend, 574804) for 7 days. All cells were incubated in a 5% CO_2_ incubator at 37 °C unless otherwise noted. All animal experiments were performed in accordance with an approved protocol from the Institutional Animal Care and Use Committee (IACUC) of RIKEN Yokohama Branch.

### Activator screen in THP-1 macrophages

THP-1 cells were seeded in tissue-culture coated 96-well plates (Corning, 3596) at a density of 100,000 cells per well in 100 μL of THP-1 media supplemented with 100 nM of phorbol-myristate acetate (PMA; Sigma, P8139), and incubated for 3 h. The media was replaced with THP-1 media without PMA and the cultures were allowed to sit overnight in CO_2_ incubator. Addition of PMA differentiates THP-1 cells from monocytes into macrophage-like cells and also primes (signal 1) to upregulate genes necessary for NLRP3 inflammasome activation. All “seeding” procedures for THP-1 cell described in the methods refer to a 3-hour incubation in 100 nM PMA/THP-1 media followed by overnight incubation in fresh THP-1 media. On the day of the screen, cells were washed once with warm PBS (Thermo, 10010-023) and chemicals from the LOPAC library were added to OPTI-MEM (Thermo, 31985-070) at 40 μM concentration and a 50 μL volume was transferred to the cell culture plate. An equal concentration of DMSO in OPTI-MEM was used as a negative control. After a 6-hour incubation, supernatants were collected and assayed for IL-1β release using a human IL-1β ELISA kit (Thermo, 88-7261-77) according to manufacturer’s protocol. Unnormalized IL-1β release level was compared across all screening plates as all of the screen was performed in a single batch.

### Suppressor screen in THP-1 macrophages

To screen for suppressors of niclosamide- and nigericin-induced IL-1β release, cells were seeded as in the activator screen, washed with PBS, and pretreated for 1 h with 40 μM of the chemicals from the same LOPAC library in 50 μL of OPTI-MEM. Then 5 μL of concentrated niclosamide or nigericin was added to each well to achieve a final concentration of 5 μM niclosamide or 20 μM nigericin and incubated for 6 h. Supernatants were collected and assayed for IL-1β release using a human IL-1β ELISA kit. IL-1β level in wells pretreated with LOPAC library were compared to wells pretreated with DMSO negative control to calculate percent suppression relative to DMSO control within each plate. All suppressor treatment followed the same protocol, 1 h pretreatment followed by addition of concentrated activators.

### Calcein cell viability assay

To screen for cell toxicity of suppressors, cells were seeded as in the suppressor screen, washed with PBS, and treated for 6 h with suppressors in OPTI-MEM. After 6 h, suppressors were removed and replaced with phenol-free DMEM (Thermo, 31053028) containing 0.5 μM of calcein-AM (Thermo, C3100MP) for 1 h. Cells were then washed three times in PBS and the fluorescence signal (Ex 485 nm/Em 528 nm) was measured using a Synergy HTX multi-mode reader (BioTek).

### Activator screen in BMDM

Bone marrow-derived macrophages were seeded at 50,000 cells per well in BMDM media in tissue-culture coated 96-well plates (Corning, 3596). The next day, cells were pretreated with 200 ng mL^−1^ of ultrapure *E. coli* K12 LPS (InvivoGen, LPS-EK Ultrapure) in BMDM media for 3 h. Cells were then washed once with warm PBS and OPTI-MEM containing 40 μM concentration of chemicals from LOPAC library in a 50 μL volume were applied to BMDM for 6 h. For controls, nigericin at 10 μM was applied to control wells of each plate. IL-1β in the supernatants were detected using a mouse IL-1β ELISA kit (Thermo, 88-7013-77) according to manufacturer’s protocol. IL-1β release level of each plate was normalized to within plate nigericin control (set at 2 ng mL^−1^) as the screen was performed using three different batches of BMDM. All measurements involving BMDM described in the methods include a 3-hour incubation with LPS (‘priming’).

### Suppressor screen in BMDM

To screen for suppressors of niclosamide- and nigericin-induced IL-1β release, cells were seeded as in the activator screen and primed for 3 h with LPS in BMDM media. Cells were then washed with PBS and pretreated for 1 h with 40 μM of the chemicals from the same LOPAC library in 50 μL of OPTI-MEM. Then 5 μL of concentrated niclosamide or nigericin was added to each well to achieve a final concentration of 5 μM niclosamide or 10 μM nigericin and incubated for 6 h. Supernatants were collected and assayed for IL-1β release using a mouse IL-1β ELISA kit. IL-1β level in wells pretreated with LOPAC library were compared to wells pretreated with DMSO negative control to calculate percent suppression relative to DMSO control within each plate. All suppressor treatment followed the same protocol, 1 h pretreatment followed by addition of concentrated activators.

### IL-1β release by mouse macrophages

Mouse peritoneal and alveolar macrophages were seeded in BMDM media in tissue-culture coated 96-well plates for 3 h. Cells which did not adhere to the plates were removed and remaining cells were treated with 200 ng mL^−1^ of ultrapure *E. coli* K12 LPS in BMDM media for 3 h. Cells were then washed once with warm PBS and switched to OPTI-MEM containing NLRP3 inflammasome activators. IL-1β in the supernatants were detected using a mouse IL-1β ELISA kit.

### Generation of knockout cells

THP-1 knockout cells were generated using a lentiCRISPR_v2 system^51^ expressing both Cas9 and gene-specific single guide RNA (sgRNA). Gene-specific sgRNA were cloned into lentiCRISPR_v2 vector (Addgene, 52961) using a vector specific protocol from Addgene and the oligonucleotide pairs listed in Supplementary Table 5. Lentiviruses were generated by cotransfecting lenti-X 293 T cells (Clontech, 632180) with gene-specific lentiCRISPR_v2, pMD2.G (Addgene, 12259) and psPAX2 (Addgene, 12260) using TransIT LT1 (Mirus, MIR2300) and virus-containing supernatants were concentrated using lenti-X concentrator (Clontech, 632180). THP-1 monocytes were infected with lentivirus in THP-1 media containing 8 μg mL^−1^ polybrene (Sigma, H9268) with 1200 X g spin-infection at 30 °C for 50 min. Cells that had incorporated the lentiviral construct were selected using 2.5 μg mL^−1^ puromycin (Sigma, P9620) in THP-1 media for 2 to 3 weeks. To normalize IL-1β release level relative to cell number, knockout cells were seeded in black-walled clear bottom 96-well plates (BD Falcon, 353219) in THP-1 media. Cells were fixed with 4% paraformaldehyde (Alfa Aesar, 043368) in PBS, stained with 1 μg mL^−1^ Hoechst 33342 (Thermo, H3570) in PBS for 10 min, washed once with PBS, and then imaged at 4x using an InCell Analyzer 2000 (GE Healthcare). Cell numbers were analyzed with CellProfiler^52^ using Otsu two-class thresholding to identify cell nucleus boundary, limited to diameter of 4 to 15 pixels.

### mtDNA depletion in THP-1 cells

THP-1 cells were cultured for 4 weeks in 50 ng mL^−1^ of ethidium bromide (Sigma, E7637) and 50 μg mL^−1^ uridine (Sigma, U3003) in THP-1 media. Cells were differentiated and primed with 100 nM PMA using the same procedure as for IL-1β detection. To assess the degree of mtDNA depletion, THP-1 cells were seeded at 1 × 10^6^ cells per well in 1 mL of THP-1 media. DNA was extracted with a QIAamp DNA mini kit (Qiagen, 51304) and mitochondrial DNA copy number (mtND2) relative to nuclear genome copy number (human Alu repeat; nuAlu) was assessed by qPCR using SYBR Premix Ex TaqII (TaKaRa, RR820A) and a StepOnePlus Real-Time PCR System (Thermo). The relative abundance of each DNA was calculated from Ct values and normalized relative to 0 μg mL^−1^ ethidium bromide treated THP-1 cells. The following primer pairs were used:

mtND2, 5′-TGTTGGTTATACCCTTCCCGTACTA-3′

mtND2, 5′-CCTGCAAAGATGGTAGAGTAGATGA-3′

nuAlu, 5′-CTTGCAGTGAGCCGAGATT-3′

nuAlu, 5′-GAGACGGAGTCTCGCTCTGTC-3′

### Extracellular pH and media carbon source

Intracellular pH was altered by incubating cells in “pH adjusted buffers” containing 140 mM NaCl, 5 mM KCl, 25 mM Tris-HCl, 1.8 mM CaCl_2_, 0.9 mM MgCl_2_, 5 g L^−1^ glucose, adjusted to pH of 6.7, 7.3, 7.9 or 8.5. Cells were incubated in a non-CO_2_ 37 °C incubator. To switch the media carbon source, glucose (Sigma, G7021) or galactose (Sigma, G0750) was added to RPMI media with no glucose (Thermo, 11879-020) at a final concentration of 10 mM. This media contains 2 mM glutamine which serves as an alternative carbon source in galactose-containing media. Cells in glucose or galactose-containing RPMI were incubated in CO_2_ incubator set at 37 °C.

### Immunoblotting

THP-1 cells were seeded at 1 × 10^6^ cells per well in 1 mL of THP-1 media and BMDM were seeded at 5 × 10^5^ cells per well in 1 mL of BMDM media in 12-well plates (Corning, 353043) using the same procedure as for IL-1β detection. Cells were treated with compounds in 500 μL of OPTI-MEM and supernatants were collected and centrifuged to clear cell debris. Cell lysates were collected in 100 μL of RIPA (Thermo, 89901) containing 1x protease/phosphatase inhibitors (Thermo, 78440). Proteins in the cell lysates were quantified using BCA (Thermo, 23227). For supernatants, proteins were extracted by adding 500 μL methanol and 125 μL chloroform followed by centrifugation at 14,000 × *g* for 5 min. The upper phase was removed and the remaining protein and bottom layer was washed gently with 500 μL methanol, centrifuged, supernatant was removed, and the pellet was dried. Protein samples were separated by SDS-PAGE followed by transfer to PVDF and immunoblotted.

The following primary antibodies were used; human IL-1β (Cell Signaling, 12242; 1:1,000 (v/v) dilution), human caspase-1 (Cell Signaling, 4199; 1:1,000 (v/v) dilution), mouse IL-1β (R&D, AF-401-NA; 1:1,000 (v/v) dilution), mouse caspase-1 (Santa Cruz, sc-514; 1:1,000 (v/v) dilution), human and mouse beta actin (Cell Signaling, 4970; 1:2,000 (v/v) dilution). Secondary antibodies included HRP-conjugated anti-mouse IgG (Cell Signaling, 7076), anti-rabbit IgG (Cell Signaling, 7074), and anti-goat IgG (R&D, HAF019) at a 1:2,000 (v/v) dilution. Proteins were visualized by chemiluminescence using Clarity Western ECL Substrate (Bio-Rad, 170-5060) and imaged on ImageQuant LAS4000 (GE Healthcare).

### RNA isolation and quantitative PCR

A total of 5 × 10^5^ THP-1 cells per well were seeded in 24-well plates (Thermo, 142475) in 500 μL THP-1 media. Treated cells were washed once with PBS and total RNA was isolated with an RNeasy Mini Kit (Qiagen, 74106). cDNA was synthesized from 100 ng of total RNA using SuperScript VILO (Thermo, 11754-250) and the relative abundance of IL-1β mRNA compared to β-actin mRNA was assessed by qPCR using SYBR Premix Ex TaqII on a StepOnePlus Real-Time PCR System (Thermo). The following primer pairs were used:

IL-1β, 5′- AGCTACGAATCTCCGACCAC-3′

IL-1β, 5′- CGTTATCCCATGTGTCGAAGAA-3′

β-actin, 5′- CATGTACGTTGCTATCCAGGC-3′

β-actin, 5′- CTCCTTAATGTCACGCACGAT-3′

### Flow cytometric analysis

THP-1 cells were seeded at 1 × 10^6^ cells per well and BMDM were seeded at 5 × 10^5^ cells per well in 1 mL media in 12-well tissue culture treated plates using the same procedure as for IL-1β detection. For mitochondrial ROS measurement, cells were treated with activators or suppressor-activator combinations in 500 μL OPTI-MEM at the indicated concentration and time, replaced with 500 μL OPTI-MEM containing 3 μM MitoSOX (Thermo, M36008) and incubated for 30 min at 37 °C in a CO_2_ incubator. Cells were detached using TrypLE (Thermo, 12604-021) and analyzed using a FACS Calibur (BD Bioscience) with Ex 488 nm/Em 585 nm. For mitochondrial membrane potential measurement, cells were detached with TrypLE and incubated with activators in Live Cell Imaging Solution (Thermo, A14291DJ) for 15 min at 500 μL volume at 37 °C followed by incubation in 10 μg mL^−1^ of JC-1 (Thermo, T3168) in Live Cell Imaging Solution for 15 min at 37 °C. Cells were washed twice in PBS and analyzed using a FACS Calibur (BD Bioscience) for JC-1 green (Ex 488 nm/Em 530 nm) and JC-1 red aggregate (Ex 488 nm/Em 585 nm). For endosomal/lysosomal proton measurement, cells were incubated with 1 μg mL^−1^ of acridine orange (Thermo, A1301) for 15 min, washed once with PBS, and incubated with activators in OPTI-MEM for 1 h. Cells were detached with TrypLE and analyzed using a FACS Calibur (BD Bioscience) with Ex 488 nm/Em 650 nm. Gating strategies are described in Supplementary Fig. 13. All FACS data were analyzed with FlowJo (v10.1).

### LDH release

THP-1 cells were seeded at 1 × 10^6^ cells per well in tissue-culture coated 96-well plates using the same procedure as for IL-1β detection. LDH levels in supernatants were measured using a Cytotoxicity Detection Kit (LDH) (Roche, 11644793001) according to the manufacturer’s protocol.

### Intracellular ATP

NLRP3 knockout THP-1 cells were seeded at 100,000 cells per well and BMDM were seeded at 50,000 cells per well in white-walled clear-bottom 96-well plates (BD Falcon, 353377). Intracellular ATP was measured using CellTiter Glo (Promega, G7571) according to the manufacturer’s protocol.

### Intracellular lactate

NLRP3 knockout THP-1 cells were seeded at 7 × 10^6^ cells per plate in 10 cm TC treated plates. Intracellular lactate levels were quantified after activator treatment using an L-lactate assay kit (Abcam, ab65331) according to the manufacturer’s protocol. Cells treated with activators were detached with TrypLE and the cell pellet was homogenized in 100 μL of lactate assay buffer using a G26 needle and deprotenated using TCA (Abcam, ab204708).

### Intracellular pH

NLRP3 knockout THP-1 cells were seeded at 50,000 cells per well and BMDM were seeded at 50,000 cells per well in black-walled clear bottom 96-well plate (BD Falcon 353219). Cells were preloaded with 5 μM BCECF-AM (Thermo, B1170) in HBSS (Thermo, 14025-092) for 30 min at 37 °C. Cells were then treated with activators in HBSS in 100 μL per well volume and pH values were determined using calibration wells containing 15 μM nigericin in calibration buffer (135 mM KCl, 2 mM K_2_HPO_4_, 20 mM HEPES, 1.2 mM CaCl_2_, 0.8 mM MgSO_4_) adjusted to pH of 5.5, 6.5, 7.5, and 8.5. For intracellular pH measurement under different extracellular pH conditions, “pH adjusted buffers” described above were used instead of HBSS. For pH measurement under glucose or galactose as carbon source, we used “pH adjusted buffers” at pH 7.9 without glucose, supplemented with either 10 mM glucose or 10 mM galactose. The fluorescence signal (Ex 485 nm/Em 528 nm) and background (Ex 380 nm/Em 528 nm) were measured using a Synergy HTX multi-mode reader (BioTek) set to 37 °C temperature. Background was subtracted from signal and a calibration curve was used to calculate intracellular pH.

### Intracellular potassium

A total of 1 × 10^6^ NLRP3 knockout THP-1 macrophages were seeded in 12-well plates in 1 mL THP-1 media. Following activator-suppressor treatments, cells were washed with PBS twice, and cell lysates were collected in 2 mL 10% nitric acid (Wako, 149-06845). Intracellular potassium was measured using a BWB-XP flame photometer (BWB Technologies). A KCl (Sigma, 24-5550) dilution series was used to generate standard curve.

### Glycolysis rate

A total of 1 × 10^5^ THP-1 macrophages were seeded per well in XFp cell culture microplates (Agilent, 103022-100). Extracellular acidification rates (ECAR) were measured in Seahorse XF media (Agilent, 103193-100) containing 2 mM GlutaMax (Thermo, 35050061), adjusted to pH of 6.7, 7.3, 7.9, or 8.5 using a Seahorse XFp Analyzer (Agilent). Basal ECAR in absence of glucose (ECAR without active glycolysis) was subtracted from ECAR after addition of 10 mM glucose (ECAR with active glycolysis) to calculate glycolysis rate.

### Oxygen consumption rate

A total of 1 × 10^5^ THP-1 macrophages were seeded per well in XFp cell culture microplates. Oxygen consumption rates (OCR) were measured in Seahorse XF media containing 10 mM glucose, 2 mM GlutaMax, pH 7.4 using a Seahorse XFp Analyzer.

### Lysosomal membrane permeabilization

Lysosomal membrane permeabilization was assessed by cathepsin B release into the cytosol following a published protocol^53^. Briefly, 5 × 10^4^ THP-1 cells were seeded in 24-well plates and treated with activators for 30 min in OPTI-MEM. Plasma membranes were permeabilized with 12 μg mL^−1^ digitonin (Sigma, D141) in digitonin extraction buffer (250 mM sucrose, 20 mM HEPES, 10 mM KCl, 1.5 mM MgCl_2_, 1 mM EDTA, 1 mM EGTA, 0.5 mM Pefabloc, pH 7.5) on ice for 10 min and plasma and lysosomal membranes were permeabilized with 200 μg mL^−1^ digitonin in digitonin extraction buffer on ice for 10 min. Cathepsin B activity from the extract was measured in 50 mM sodium acetate, 4 mM EDTA, 8 mM DTT, 0.5 mM Pefabloc (Roche, 11873601001), 50 μM zFR-AFC (Enzo, ALX260-129-M005) using Ex 360 nm/Em 528 nm at 30 °C for 20 min using a Synergy HTX multi-mode reader (BioTek) and change in fluorescence over time was used to calculate enzyme activity. Cathepsin B activity was normalized to the extent of plasma membrane permeabilization as assessed by LDH activity. Normalized cathepsin B activity between 12 μg mL^−1^ digitonin and 200 μg mL^−1^ digitonin was used to calculate lysosomal membrane permeabilization as percent of maximum cathepsin B activity.

### In vitro caspase-1 activity assay

In vitro caspase-1 activity was assessed by modification of a published protocol^54^. A total of 1 × 10^7^ THP-1 cells were seeded in 25 cm plates in 25 mL THP-1 media containing 100 nM PMA using the same procedure as for IL-1β detection. On the next day, the cells were detached with TrypLE and lysed in ice-cold hypotonic buffer containing 25 mM HEPES (pH 7.5), 5 mM MgCl_2_, 1.3 mM EDTA, 1 mM EGTA, 1 mM PMSF (Sigma, P7626), 1x protease/phosphatase inhibitors by passing through a G26 needle 15 times, and lysate was collected after centrifugation at 16,000 X g for 30 min at 4 °C. Lysate containing 200μg of protein was placed in 200 μL of caspase reaction buffer containing 25 mM HEPES, 2 mM DTT, 50 μM Ac-YVAD-AMC (Enzo, ALX-260-024-M005), adjusted to the different pH values. Caspase-1 activity was detected at Ex 380 nm/Em 460 nm every 1 min for 30 min using a Synergy HTX multi-mode reader set to 37 °C temperature and change in raw fluorescence signal (RFU) over 30 min interval was used to derive relative enzyme activity (RFU per min).

### Statistical analysis

All data are presented from one independent experiment, representative of at least two independent experiments unless otherwise noted. Statistical significance was assessed using an unpaired, two-tailed Student’s *t* test and one-way or two-way ANOVA followed by Tukey’s multiple testing in Prism6 (GraphPad). All the data are assumed normally distributed. Variance could not be determined due to small sample sizes.

## Supplementary information

Description of  Additional Supplementary Files

Supplementary Information

Supplementary Data 2

Supplementary Data 1

## Data Availability

CellProfiler pipeline and chemical screening data are available on dryad^55^. Full immunoblot images are available in Supplementary Figures 14 and 15. All other dataset generated or analyzed are available from the corresponding author upon request.
