# Supplementary material for: Niclosamide activates the NLRP3 inflammasome by intracellular acidification and mitochondrial inhibition
Source: Commun Biol. 2019 Jan 3;2:2. doi: 10.1038/s42003-018-0244-y (PMC6318214; doi:10.1038/s42003-018-0244-y)
Supplement: Supplementary file 1 — Description of Additional Supplementary Files [file 42003_2018_244_MOESM1_ESM.docx]

**Description of Additional Supplementary Files**

**File Name**: Supplementary Data 1

**Description**: Results of activator and suppressor screens in human THP-1 macrophages across 1280 compounds (Excel file).

Data analysis schemes are described in Supplementary Figure 1.

**Excel Sheet 1 (PrimaryScreens):**

- Column G (Activator Screen at 40μM) refers to IL-1β release level after 6h treatment with 40μM of screening compounds.
- Column H (Suppressor screen at 40μM: niclosamide stimulated) refers to IL-1β release level after 1h pretreatment with 40μM of screening compounds, followed by 6h niclosamide treatment at 5μM concentration in the presence of screening compounds. Data are presented as % of IL-1β release relative to in-plate DMSO negative control. 0% means complete suppression of IL-1β release.
- Column I (Suppressor screen at 40μM: nigericin stimulated) refers to IL-1β release level after 1h pretreatment with 40μM of screening compounds, followed by 6h nigericin treatment at 20μM concentration in the presence of screening compounds. Data are presented as % of IL-1β release relative to in-plate DMSO negative control. 0% means complete suppression of IL-1β release.

**Excel Sheet 2 (ActivatorValidation):**

- Columns G-J (Activator Validation) refer to IL-1β release levels after 6h treatment with top 132 activators (from THP-1 screen) at four indicated concentrations.
- Column K (IL-1β @ 40μM in NLRP3_sgRNA cell) refers to % of IL-1β release level in NLRP3 knockout cells (expressing NLRP3 sgRNA) relative to control cells (expressing N.Ctl1 sgRNA) after 6h treatment with top activators. 0% means complete suppression of IL-1β release in NLRP3 knockout relative to control cell.
- Column L (IL-1β @ 40μM in CASP1_sgRNA cell) refers to % of IL-1β release level in CASP1 knockout cells (expressing CASP1 sgRNA) relative to control cells (expressing N.Ctl1 sgRNA) after 6h treatment with top activators. 0% means complete suppression of IL-1β release in CASP1 knockout relative to control cells.

**Excel Sheet 3 (SuppressorValidationNiclosamide):**

- Columns G-J (Suppressor Validation: niclosamide stimulated) refer to % of IL-1β release level after 1h pretreatment with top 80 suppressors (of niclosamide-induced IL-1β release) at indicated concentrations followed by 6h treatment with 5μM niclosamide in the presence of suppressors. Data are presented as % of IL-1β release relative to in-plate DMSO negative control.
- Columns K-N (Calcein cell viability) refer to % of calcein-AM fluorescence after 6h treatment with top 80 suppressors at indicated concentrations. Data are presented as % of calcein-AM signal relative to in-plate DMSO negative control.

**Excel Sheet 4 (SuppressorValidationNigericin):**

- Columns G-J (Suppressor Validation: nigericin stimulated) refer to % of IL-1β release level after 1h pretreatment with top 80 suppressors (of nigericin-induced IL-1β release) at indicated concentrations followed by 6h treatment with 20μM nigericin in the presence of suppressors. Data are presented as % of IL-1β release relative to in-plate DMSO negative control.
- Columns K-N (Calcein cell viability) refer to % of calcein-AM fluorescence after 6h treatment with top 80 suppressors at indicated concentrations. Data are presented as % of calcein-AM signal relative to in-plate DMSO negative control.

**File Name**: Supplementary Data 2

**Description**: Results of activator and suppressor screens in LPS-primed mouse bone marrow derived macrophages across 1280 compounds (Excel file).

Data analysis schemes are described in Supplementary Figure 1.

**Excel Sheet 1 (PrimaryScreens):**

- Column G (Activator Screen at 40μM) refers to IL-1β release level after 6h treatment with 40μM of screening compounds. IL-1β release level is normalized to in-plate nigericin control (10μM) set at 2000pg mL^-1^.
- Column H (Suppressor screen at 40μM: niclosamide stimulated) refers to IL-1β release level after 1h pretreatment with 40μM of screening compounds, followed by 6h niclosamide treatment at 5μM concentration in the presence of screening compounds. Data are presented as % of IL-1β release relative to in-plate DMSO negative control. 0% means complete suppression of IL-1β release.
- Column I (Suppressor screen at 40μM: nigericin stimulated) refers to IL-1β release level after 1h pretreatment with 40μM of screening compounds, followed by 6h nigericin treatment at 10μM concentration in the presence of screening compounds. Data are presented as % of IL-1β release relative to in-plate DMSO negative control. 0% means complete suppression of IL-1β release.

**Excel Sheet 2 (ActivatorValidation):**

- Columns G-J (Activator Validation) refer to IL-1β release levels after 6h treatment with top 80 activators (from BMDM screen) at four indicated concentrations. IL-1β release level is normalized to in-plate nigericin control (10μM) set at 2000pg mL^-1^.

**Excel Sheet 3 (SuppressorValidationNiclosamide):**

- Columns G-J (Suppressor Validation: niclosamide stimulated) refer to % of IL-1β release level after 1h pretreatment with top 80 suppressors (of niclosamide-induced IL-1β release in BMDM) at indicated concentrations followed by 6h treatment with 5μM niclosamide in the presence of suppressors. Data are presented as % of IL-1β release relative to in-plate DMSO negative control.

**Excel Sheet 4 (SuppressorValidationNigericin):**

- Columns G-J (Suppressor Validation: nigericin stimulated) refer to % of IL-1β release level after 1h pretreatment with top 80 suppressors (of nigericin-induced IL-1β release in BMDM) at indicated concentrations followed by 6h treatment with 10μM nigericin in presence of suppressors. Data are presented as % of IL-1β release relative to in-plate DMSO negative control.

**Excel Sheet 5 (SuppressorValidationBiased):**

- Columns G-J (Suppressor Validation: niclosamide stimulated) refer to % of IL-1β release level after 1h pretreatment with top 36 suppressors (of niclosamide-induced IL-1β release but not of nigericin-induced IL-1β release in BMDM) at indicated concentrations followed by 6h treatment with 5μM niclosamide or 10μM nigericin in the presence of suppressors. Data are presented as % of IL-1β release relative to in-plate DMSO negative control. A separate validation screen from sheet 3 and 4 were performed for BMDM because many of the top suppressors of niclosamide-induced IL-1β release overlapped with top suppressors of nigericin-induced IL-1β release. These top 36 suppressors showed >80% suppression of niclosamide-induced IL-1β release and <20% suppression of nigericin-induced IL-1β release in primary screen (Sheet 1).
