# Supplementary material for: Niclosamide activates the NLRP3 inflammasome by intracellular acidification and mitochondrial inhibition
Source: Commun Biol. 2019 Jan 3;2:2. doi: 10.1038/s42003-018-0244-y (PMC6318214; doi:10.1038/s42003-018-0244-y)
Supplement: Supplementary file 2 — Supplementary Information [file 42003_2018_244_MOESM2_ESM.pdf]

Supplementary Figures

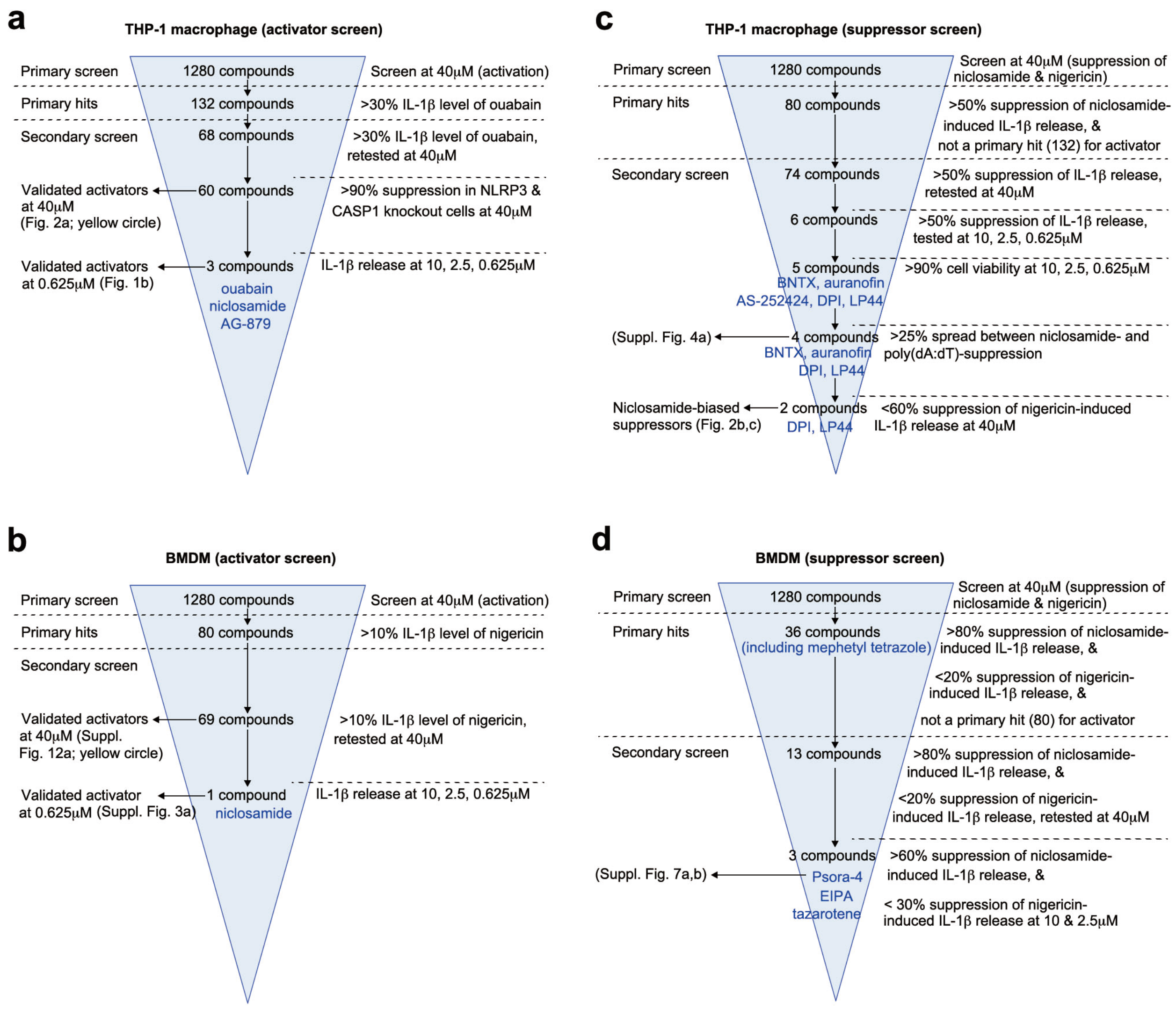

Supplementary Figure 1. Schematics of hit selection for activator and suppressor screens in THP-1 macrophages and BMDMs. (**a**) Criteria for activator hit selection in THP-1 macrophages. (**b**) Criteria for activator hit selection in bone marrow derived macrophages (BMDM). (**c**) Criteria for suppressor hit selection in THP-1 macrophages. (**d**) Criteria for suppressor hit selection in BMDM. Nigericin was added as a reference for activator screen in BMDM because ouabain at 40μM screening concentration does not achieve robust IL-1β release in BMDM. For niclosamide-biased suppressors in BMDM, because all top 80 suppressors of niclosamide-induced IL-1β release were also suppressors of nigericin-induced IL-1β release, we used both niclosamide and nigericin suppressor screen data for primary hit selection. Some of the hit compounds mentioned in the main text are indicated in blue.

a

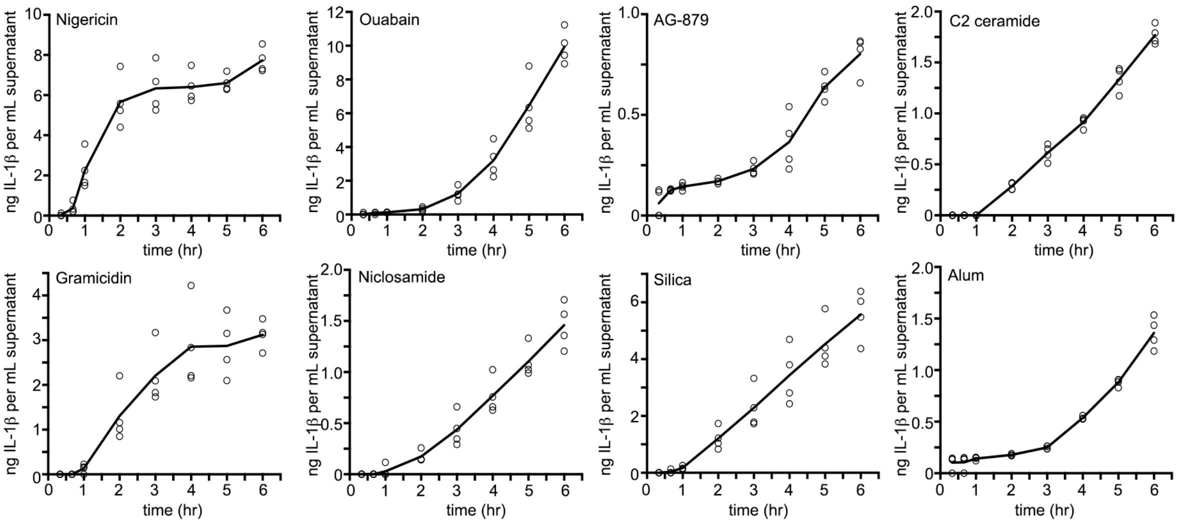

Supplementary Figure 2. Time course analysis of IL-1β release from THP-1 macrophages. (**a**) IL-1β release level after treatment with activators at indicated time in THP-1 macrophages. Data are n = 4 biological replicates. Activators were used at 20μM (nigericin), 10μM (gramicidin), 1μM (ouabain), 1μM (niclosamide), 2.5μM (AG-879), 250μg mL^{-1} (silica), 125μM (C2 ceramide), or 500μg mL^{-1} (alum).

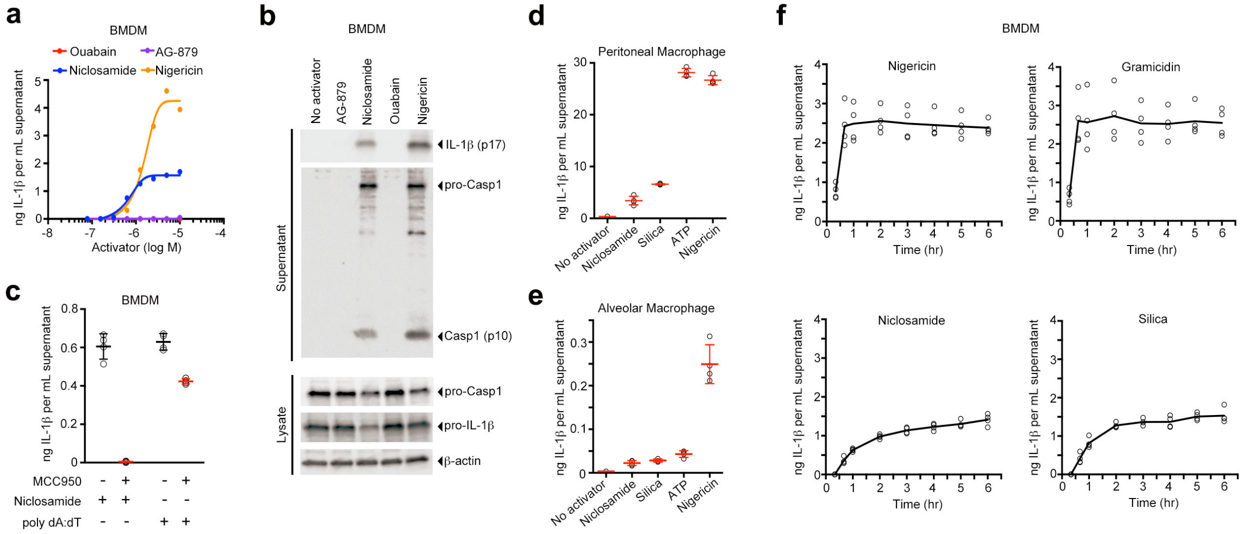

Supplementary Figure 3. NLRP3 inflammasome activation by niclosamide in mouse macrophages. (**a**) IL-1β released from LPS-primed BMDM following 6h treatment with niclosamide, AG-879, ouabain, or nigericin at the indicated concentrations. Each dot corresponds to a single biological replicate; data are representative of two independent experiments. (**b**) Immunoblot of biologically active IL-1β (p17) and caspase-1 (Casp1; p10) after 6h treatment with activators in LPS-primed BMDM. Data are representative of two independent experiments. Full gel images are shown in **Supplementary Figure 14**. (**c**) IL-1β released from LPS-primed BMDM pretreated for 1h with NLRP3 inflammasome inhibitor MCC950 followed by 6h incubation with niclosamide or poly(dA:dT) in the presence of MCC950. (**d,e**) IL-1β released from LPS-primed mouse peritoneal (**d**) and alveolar (**e**) macrophage following 6h treatment with activators. (**f**) IL-1β released from LPS-primed BMDM following activator treatment at indicated time points. (**c,d-f**) Data are n = 4 biological replicates from one independent experiment; data are representative of two independent experiments. Mean ± s.d. are shown (**c,d,e**). (**b-f**) Activators were used at 5μM (niclosamide), 10μM (AG-879), 10μM (ouabain), 10μM (nigericin), 10μM (gramicidin), 250μg mL^{-1} (silica), 5mM (ATP), 10μg mL^{-1} (poly dA:dT), and MCC950 was used at 2.5μM.

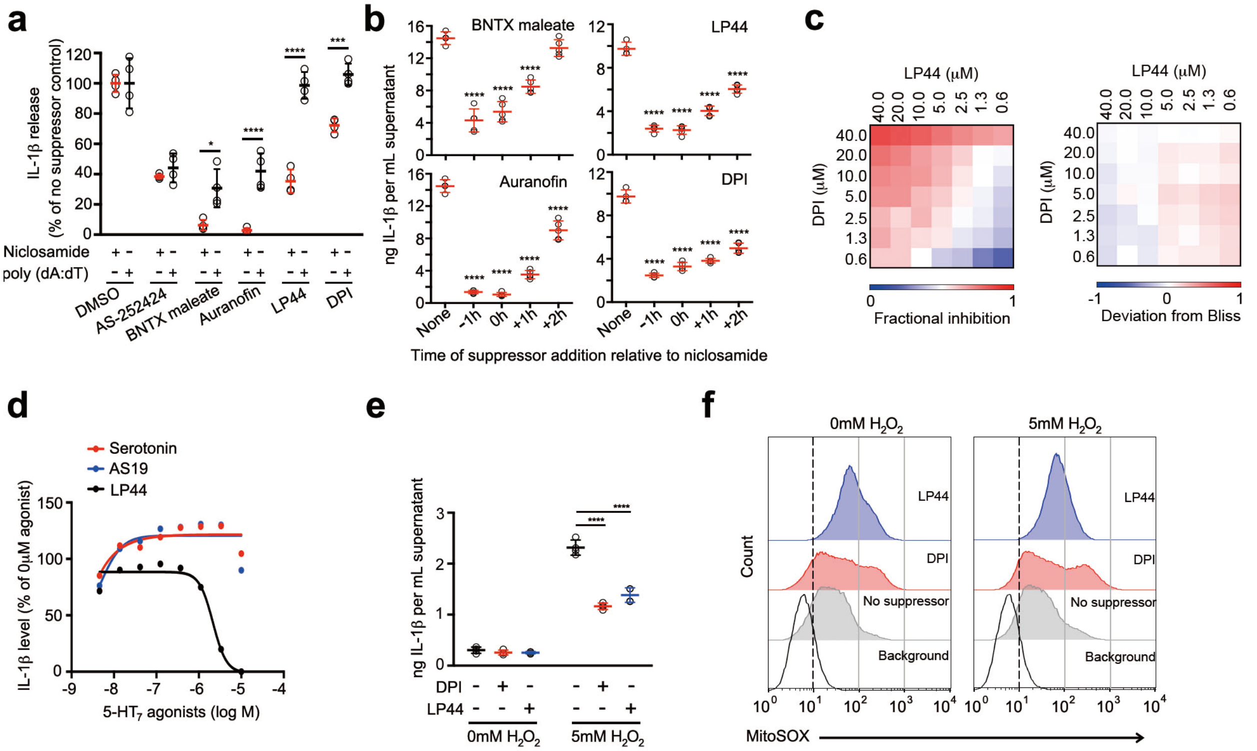

Supplementary Figure 4. Characterization of niclosamide-biased suppressors in THP-1 macrophages. (**a**) Effect of top 5 niclosamide suppressors on niclosamide- and poly(dA:dT)-induced IL-1β release. (**b**) Effect of timing of suppressor addition on IL-1β release level. (**c**) Effect of combinatorial treatment of DPI and LP44 on niclosamide-induced IL-1β release and deviation from Bliss independence model. Heat map was generated using average of 3 biological replicates. (**d**) Effect of 5-hydroxytryptamine (serotonin) receptor 7 (5-HT$_7$) agonists on niclosamide-induced IL-1β release. Each dot corresponds to a single biological replicate. (**e-f**) Effect of DPI and LP44 on hydrogen peroxide-induced IL-1β release (**e**) and mitochondrial ROS (**f**). (**a,b,e**) Data are mean ± s.d.; n = 4 (**a**,**e**), or 5 (**b**) biological replicates from one independent experiment; data are representative of two independent experiments. *P* values were determined by one-way (**b**) or two-way (**a**,**e**) ANOVA followed by Tukey's multiple testing. **P* < 0.05, ****P* < 0.001, *****P* < 0.0001 compared to no suppressor control (**b**) or as indicated (**a**,**e**). 1h suppressor treatment (**a-f**) was followed by 6h niclosamide (1μM) (**a-d**), 6h poly(dA:dT) (10μg mL^{-1}) (**a**), or 6h hydrogen peroxide (5mM) (**e-f**) treatment. Suppressors were used at (**a**) 2.5μM (AS-252424, BNTX maleate, auranofin, DPI, LP44), (**b**) 2.5μM (BNTX maleate, auranofin), or (**b**,**e**,**f**) 10μM (DPI, LP44) concentrations. THP-1 macrophages (**a-e**) or THP-1 macrophages expressing *NLRP3* guide RNA (**f**) were used for measurements.

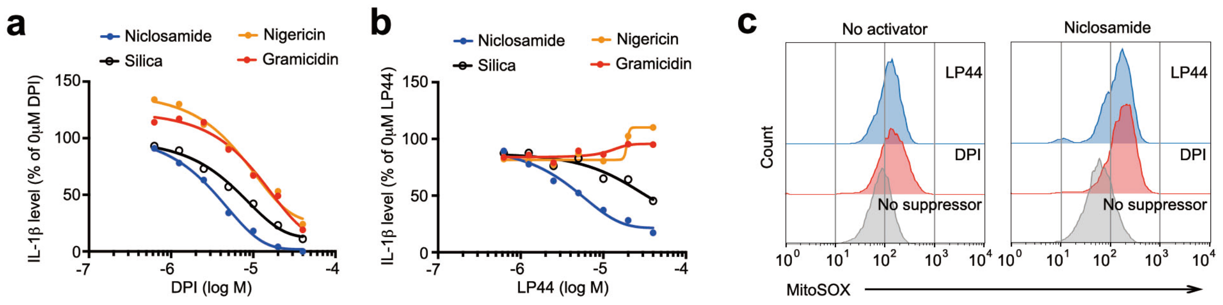

Supplementary Figure 5. Characterization of DPI and LP44 in BMDMs. (**a**,**b**) Effect of niclosamide-biased suppressors DPI and LP44 on IL-1β release in LPS-primed BMDM. Each dot corresponds to a single biological replicate; data are representative of two independent experiments. (**c**) FACS analysis of mitochondrial ROS in LPS-primed BMDM. Data represent two independent experiments. Activators were used at 5μM niclosamide (**a**-**c**), 10μM nigericin (**a**,**b**), 10μM gramicidin (**a**,**b**), 250μg mL^{-1} silica (**a**,**b**), for 1h (**c**) or 6h (**a**,**b**) following 1h pretreatment with 20μM DPI (**c**), 20μM LP44 (**c**), or as indicated (**a**,**b**).

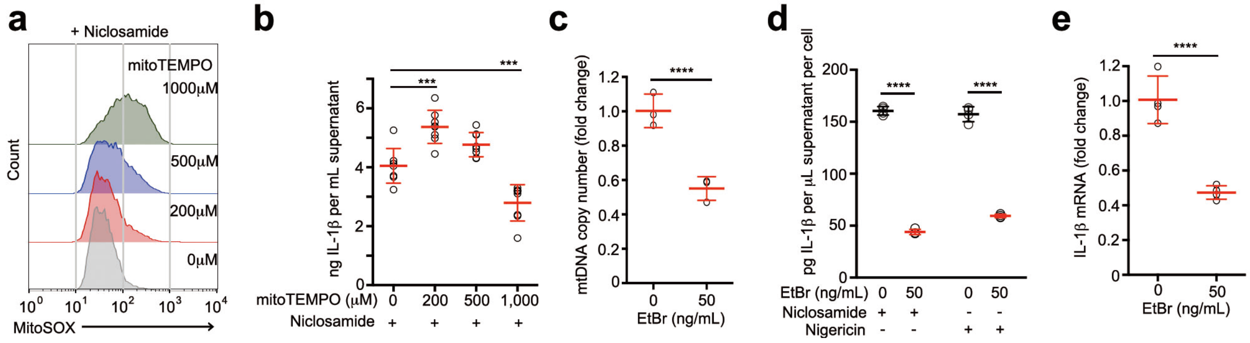

Supplementary Figure 6. Effect of mitochondrial ROS scavenger and mitochondrial DNA depletion on niclosamide-induced IL-1β release from THP-1 macrophages. (**a**) Mitochondrial ROS measurement in THP-1 macrophages after 1h pretreatment with mitoTEMPO followed by 1h treatment with niclosamide (1μM). Data represent two independent experiments. (**b**) IL-1β released from THP-1 macrophages after 1h pretreatment with mitoTEMPO followed by 6h treatment with niclosamide (1μM). (**c**) Changes in mitochondrial DNA (mtDNA) copy number in THP-1 macrophages after 4-week treatment with ethidium bromide (EtBr; 50ng mL^{-1}). (**d**) IL-1β released from EtBr-treated THP-1 macrophages after 6h treatment with niclosamide (1μM) or nigericin (20μM). (**e**) IL-1β mRNA level in EtBr-treated THP-1 macrophages. Data are mean ± s.d.; n = 3 (**c**), 4 (**d**), or 8 (**b**,**e**) biological replicates from one independent experiment; data are representative of two independent experiments. *P* values were determined by two-tailed Student's *t*-test (**c**, **e**) or by one-way (**b**) and two-way (**d**) ANOVA followed by Tukey's multiple testing. ***$P < 0.001$, ****$P < 0.0001$.

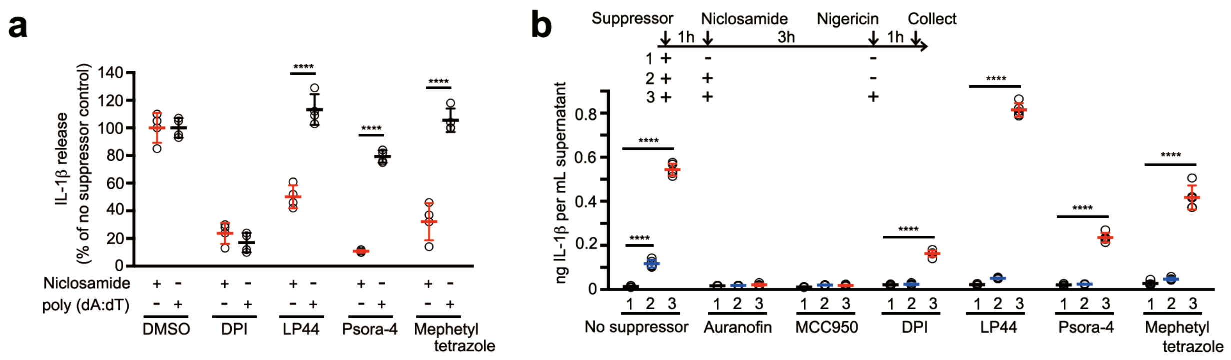

Supplementary Figure 7. Characterization of niclosamide-biased suppressors in BMDMs. (**a**) Effect of niclosamide-biased suppressors on niclosamide- and poly(dA:dT)-induced IL-1β release in LPS-primed BMDM. (**b**) Rescue of IL-1β release by nigericin (1h at 10μM) following suppression of niclosamide-induced IL-1β release in LPS-primed BMDM. Numbers (1-3) represent experimental scheme illustrated above the figure panel. Activators were used at 5μM niclosamide (**a**,**b**) or 10μg mL^{-1} poly(dA:dT) (**a**) for 6h following 1h pretreatment with 20μM DPI, 20μM LP44, 2.5μM auranofin, 2.5μM MCC950, 10μM Psora-4, or 10μM mephetyl tetrazole. Data are mean ± s.d.; n = 4 (**a**), or 5 (**b**) biological replicates from one independent experiment; data are representative of two independent experiments. *P* values were determined by two-way ANOVA followed by Tukey's multiple testing. ****$P < 0.0001$.

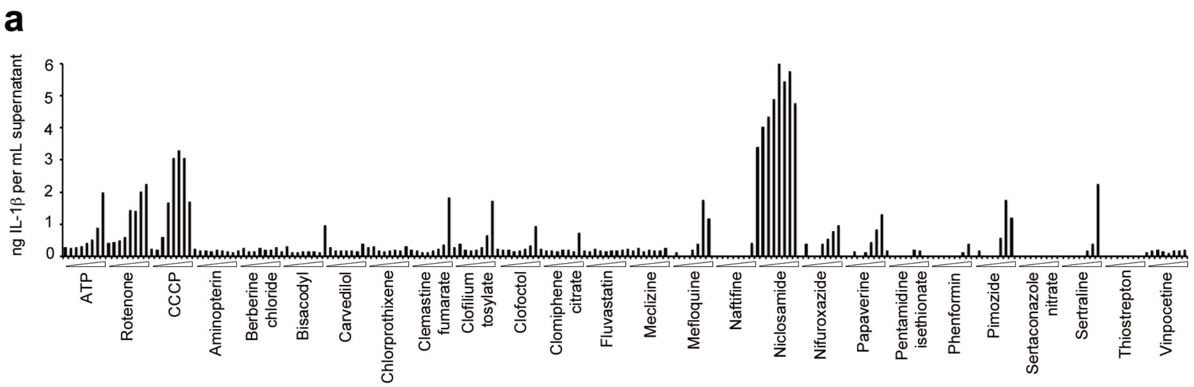

Supplementary Figure 8. Focused screen of mitochondrial inhibitors for IL-1β release from THP-1 macrophages. (**a**) IL-1β release from THP-1 macrophages following 6h treatment with compounds previously identified to possess inhibitory activity against mitochondrial respiration. Each compound consists of 8-point dilution series (two-fold dilution) starting at 40μM concentration. Each bar represents single biological replicate. For comparison, extracellular ATP starting with 10mM concentration was applied (2-fold dilution series).

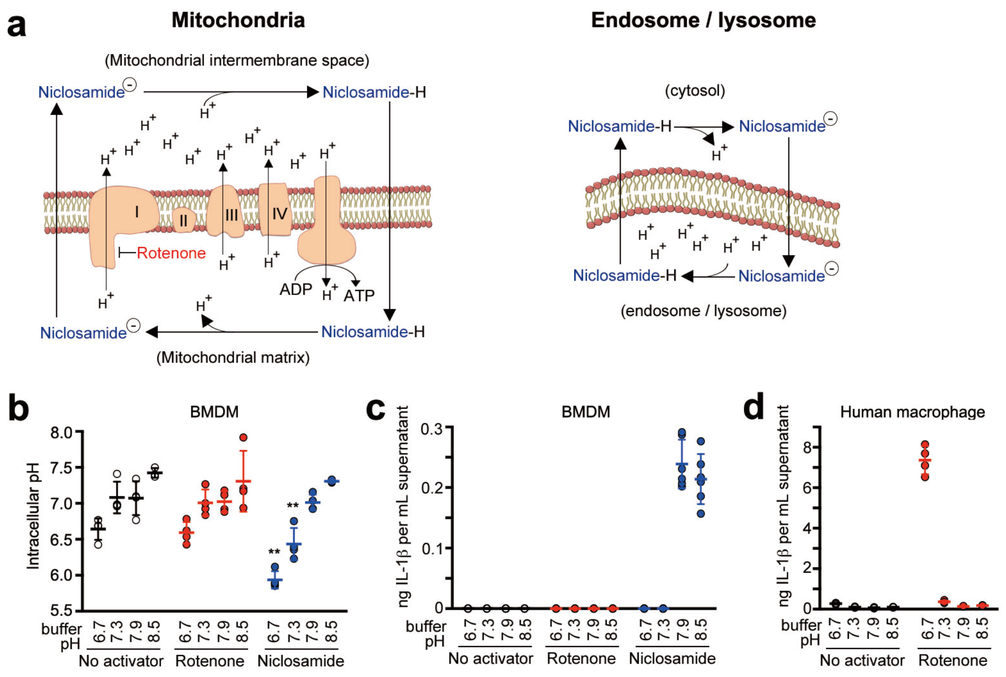

Supplementary Figure 9. Effect of extracellular pH on IL-1β release from BMDM and human macrophage. (**a**) Schematic of the different sites of action between two mitochondrial inhibitors, rotenone and niclosamide. (**b**) Intracellular pH in LPS primed BMDM treated for 1h with rotenone (5μM) or niclosamide (5μM) using buffer adjusted to different pH values. Data are mean ± s.d.; n = 4 biological replicates from one independent experiment; data are representative of two independent experiments. *P* values were determined by two-way ANOVA followed by Tukey's multiple testing. ** P < 0.01 compared to no activator control for each buffer pH. (**c**) IL-1β released from LPS-primed BMDM after 6h treatment with rotenone (5μM) or niclosamide (5μM) using buffers adjusted to different pH values. Data are mean ± s.d.; n = 6 biological replicates from one independent experiment; data are representative of two independent experiments. (**d**) IL-1β released from LPS-primed human $CD14^+$ monocyte derived macrophages after 6h treatment with rotenone (5μM) using buffers adjusted to different pH values. Data are mean ± s.d.; n = 4 biological replicates from one independent experiment.

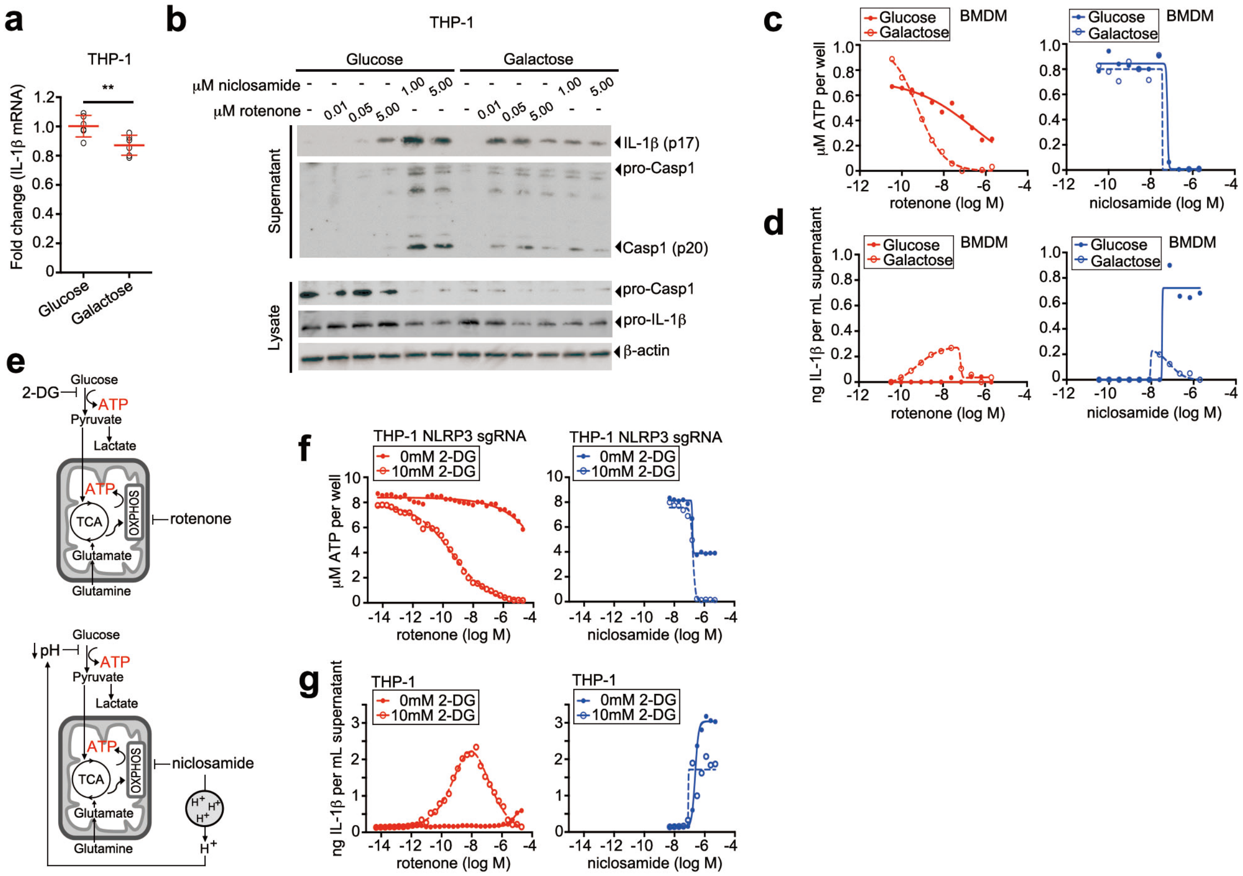

Supplementary Figure 10. Effect of fuel source on IL-1β release in BMDM and THP-1 macrophages. (**a**) *IL-1β* mRNA levels in THP-1 macrophages after 6h incubation with glucose- or galactose-containing media. Data are mean ± s.d.; n = 6 biological replicates from one independent experiment; data are representative of two independent experiments. *P* values were determined by two-tailed Student's *t*-test. $^{**}P < 0.01$. (**b**) Immunoblot of biologically active IL-1β (p17) and caspase-1 (Casp1; p20) in THP-1 macrophages after 6h treatment with rotenone or niclosamide using glucose- or galactose-containing media. Data are representative of two independent experiments. Full gel images are shown in **Supplementary Figure 15**. (**c**,**d**) Intracellular ATP levels (**c**) and IL-1β release level (**d**) in LPS-primed BMDM after 6h treatment with varying concentrations of rotenone or niclosamide using glucose- or galactose-containing media. Each dot corresponds to a single experimental replicate; data are representative of two independent experiments. (**e**) Schematic of targets for 2-deoxyglucose (2-DG), rotenone, and niclosamide with respect to ATP synthesis. (**f**,**g**) Effect of 2-deoxyglucose on intracellular ATP level (**f**) and IL-1β release (**g**) in THP-1 macrophages. ATP measurement was performed in THP-1 macrophages expressing *NLRP3* guide RNA (sgRNA) (**f**). Each dot corresponds to a single experimental replicate; data are representative of two independent experiments.

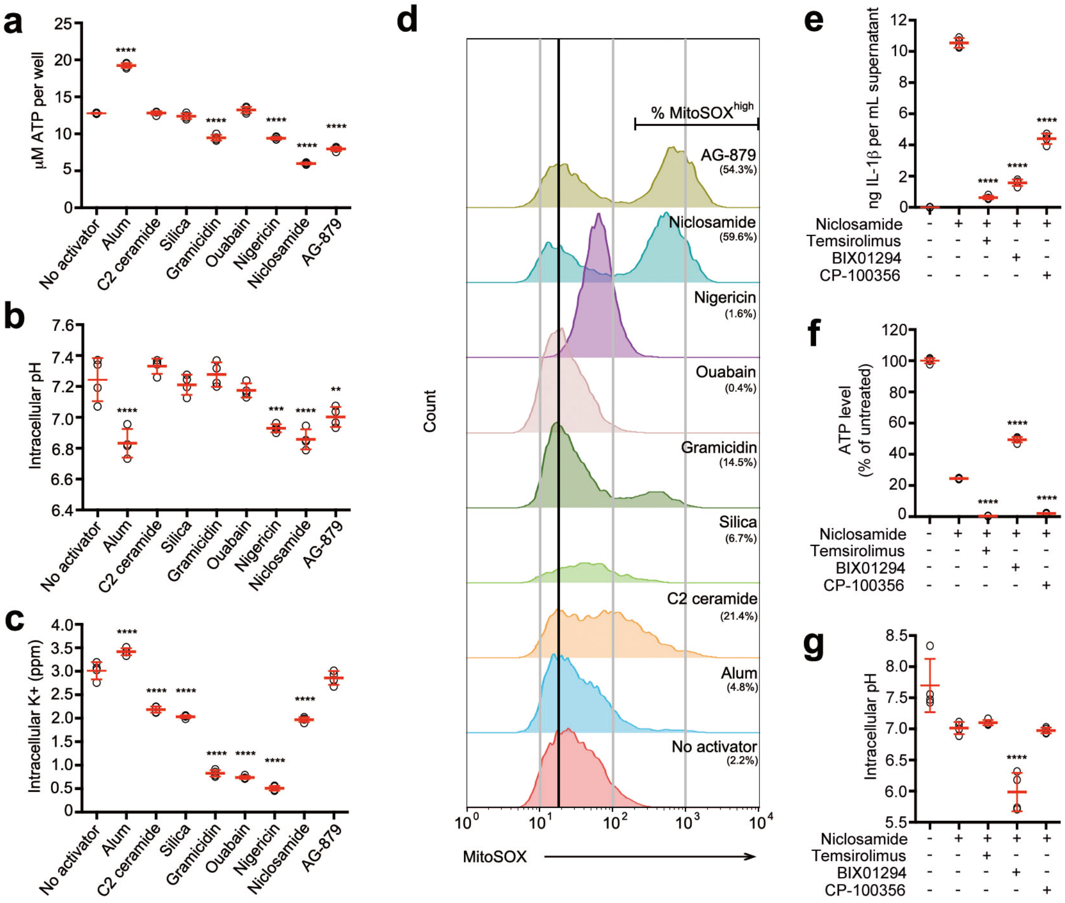

Supplementary Figure 11. Individual graphs of data summarized in Fig. 5a and 6a-b.
(**a-d**) Intracellular ATP (**a**), intracellular pH (**b**), intracellular potassium (**c**), and mitochondrial ROS (**d**) in THP-1 macrophages expressing *NLRP3* guide RNA after treatment with alum (500μg mL^{-1}), C2 ceramide (125μM), silica (250μg mL^{-1}), gramicidin (10μM), ouabain (1μM), nigericin (20μM), niclosamide (1μM), or AG-879 (2.5μM). Cells were treated for 1h (**a**, **b**, **d**) or 6h (**c**). (**d**) Percent of cells within the MitoSOX^{high} gate are indicated in parentheses. (**e-g**) IL-1β release (**e**), intracellular ATP (**f**), and intracellular pH (**g**) in THP-1 macrophages expressing negative control (N.Ctl1) guide RNA (**e**) or *NLRP3* guide RNA (**f-g**). Cells were pretreated for 1h with 40μM temsirolimus, BIX01294, or CP-100356 followed by 6h treatment (**e-f**) or 1h treatment (**g**) with niclosamide (1μM). Data are mean ± s.d.; n = 4 biological replicates from one independent experiment (**a-c**, **e-g**). Data are representative of two independent experiments (**a-g**). *P* values were determined by one-way ANOVA followed by Tukey's multiple testing. ***P* < 0.01, ****P* < 0.001, *****P* < 0.0001 compared to no activator control (**a-c**) or cells treated only with niclosamide (no second activator) (**e-g**).

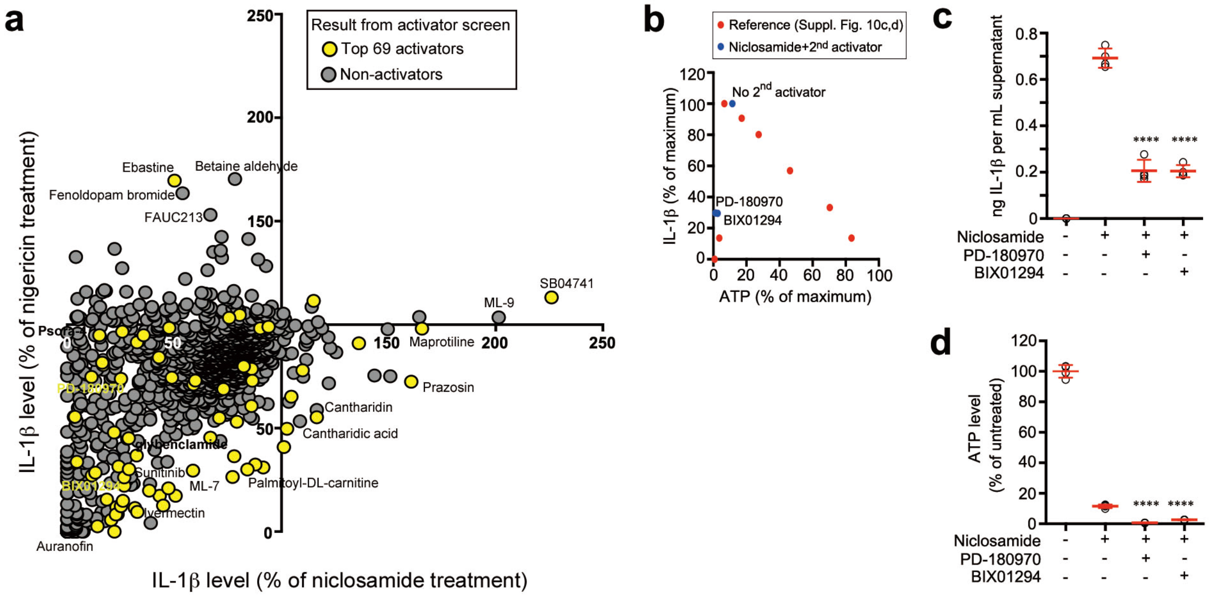

Supplementary Figure 12. Antagonism between two activators in BMDM. (**a**) Results of suppressor screens in BMDM using nigericin (10μM) or niclosamide (5μM) as activators. Yellow circles indicate the top 69 validated activators. (**b**) Relationship between intracellular ATP levels and IL-1β release in LPS-primed BMDM. The reference curve was drawn using data from **Supplementary Fig. 10c,d** (rotenone treatment in galactose). Blue dots indicate an antagonistic interaction between niclosamide and a second activator in (**a**; yellow circle). (**c**) Suppression of IL-1β release by combining niclosamide with a second activator. (**d**) A severe decline in intracellular ATP level after combining niclosamide with a second activator. (**c**,**d**) Cells were pretreated for 1h with second activators (40μM PD-180970 or 40μM BIX01294) followed by 6h treatment with 5μM niclosamide in the presence of second activator. (**c**,**d**) Data are mean ± s.d.; n = 4 biological replicates from one independent experiment; data are representative of two independent experiments. *P* values were determined by one-way ANOVA followed by Tukey's multiple testing. ****$P < 0.0001$ compared to cells treated only with niclosamide (without second activator).

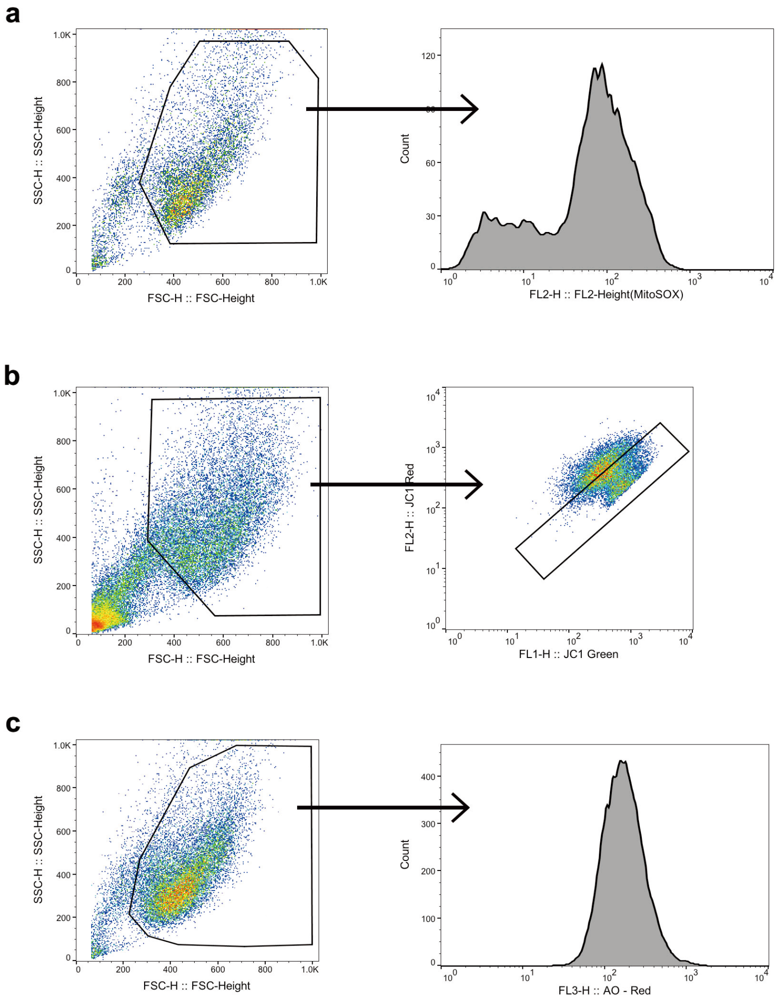

Supplementary Figure 13. Gating strategy for FACS analysis. (**a**) Gating strategy for MitoSOX measurement. (**b**) Gating strategy for JC1 measurement. Untreated cells were used to define a boxed gate for JC1 green versus JC1 red plot. (**c**) Gating strategy for acridine orange measurement. All examples are from THP-1 macrophages following niclosamide treatment.

Figure 1c

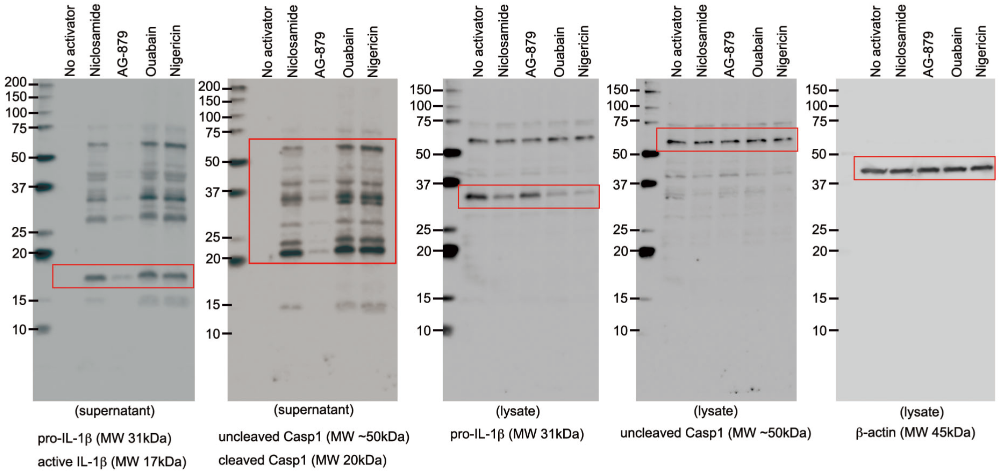

Suppl. Figure 3b

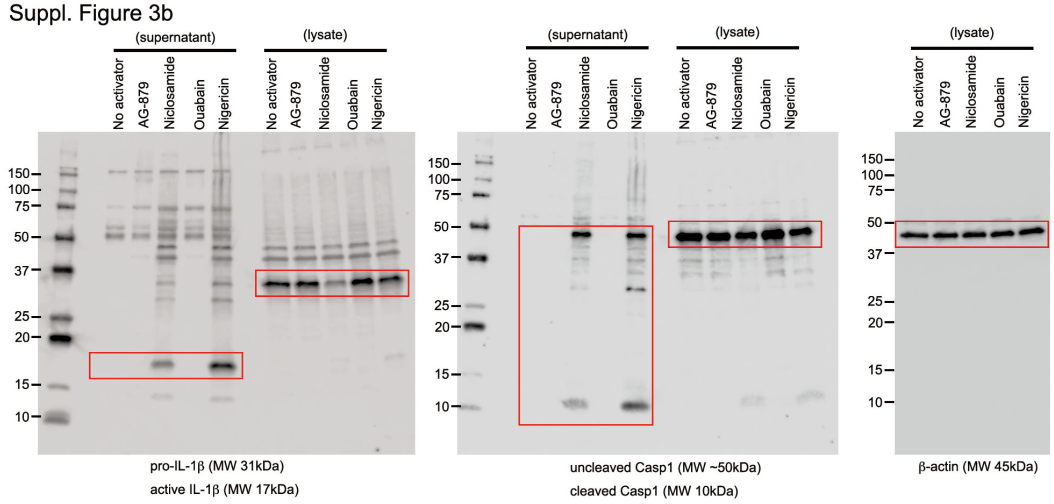

Figure 2e

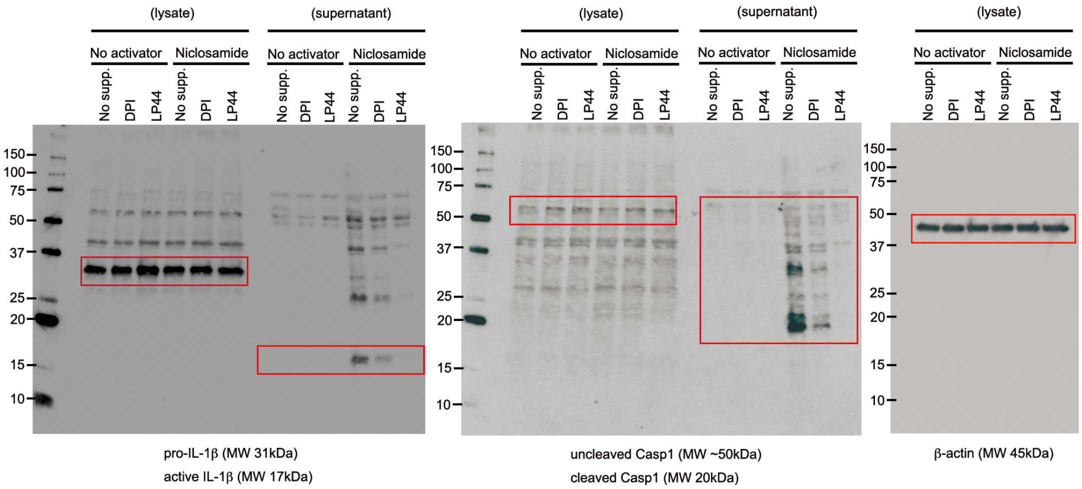

Supplementary Figure 14. Full images of immunoblots.

Supplementary Figure 10b

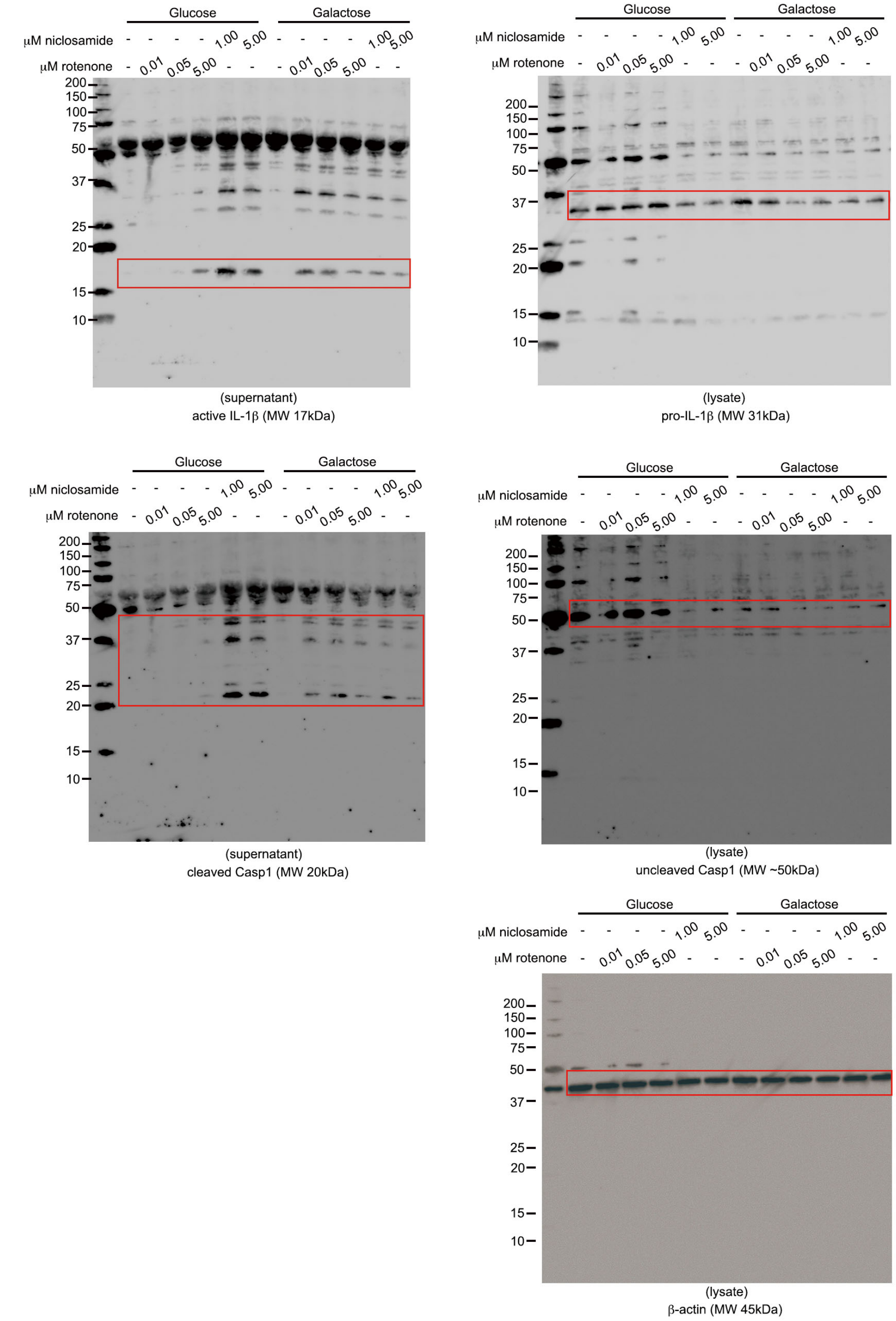

Supplementary Figure 15. Full images of immunoblots.

Supplementary Table 1. Primary screen for activators of IL-1β release in THP-1 macrophages.

Category	Parameter	Description
Assay	Type of assay	Unbiased phenotypic screen for small molecules that induce IL-1β release in THP-1 macrophage
	Target	Not applicable
	Primary measurement	IL-1β level in cell culture supernatant quantified by ELISA
	Key reagents	Human IL-1β ELISA kit (Thermo; 88-7261-77)
	Assay protocol	See Methods
Library	Nature of the library	1,280 bioactives
	Size of the library	1,280 compounds arrayed in 96-well format as single compounds at 10mM in DMSO
	Source	Sigma LOPAC library (Sigma; LO4200)
	Concentration tested	40mM, 0.4% v/v DMSO
Screen	Format	96-well plates
	Plate controls	Negative control = DMSO
	Reagent/compound dispensing system	THP-1 cells were dispensed using P300 multichannel pipette (Eppendorf) at 100μL volume. 0.6μL of compounds were dispensed into 150μL of OPTI-MEM using P10 multichannel pipette (Eppendorf) and 50μL of compounds in OPTI-MEM were transferred to cell culture plate using P300 multichannel pipette.
	Detection instrument and software	Absorbance reading at 540nm and 450nm using iMARK microplate reader (Bio-Rad).
	Assay validation/QC	IL-1β release below background level for DMSO negative control
	Correction factors	Uncorrected (single replicate)
	Normalization	Unnormalized (absolute quantitation of IL-1β level)
Post-HTS analysis	Hit criteria	IL-1β release level greater than 30% of the value of ouabain (40μM) treatment, corresponding to top 132 compounds
	Hit rate	10.3% (132/1280)
	Retesting of initial actives	Top 132 compounds based on IL-1β release level were rearrayed and retested at 40μM, 10μM, 2.5μM, and 0.625μM concentration; the same 132 compounds were tested for IL-1β release level in NLRP3 and CASP1 knockout cells at 40μM concentration; compounds with IL-1β release level >30% of ouabain at 40μM concentration and suppressible in NLRP3 and CASP1 knockout cells (>90% suppression) were labeled 'validated activators' (60 compounds); of these, compounds that maintained IL-1β release at 10μM, 2.5μM, and 0.625μM concentration were retested in dose-response mode (3 compounds)
	Confirmation of hit purity and structure	Validated hits (3 compounds) were repurchased and retested (Fig. 1b)
Screen results	List of all screening positives	List of IL-1β release level at 40μM concentration (Supplementary Data 1; PrimaryScreens)
	List of validated compounds	List of IL-1β release level at 4 concentrations (Supplementary Data 1; ActivatorValidation)

Supplementary Table 2. Primary screen for activators of IL-1β release in BMDM.

Category	Parameter	Description
Assay	Type of assay	Unbiased phenotypic screen for small molecules that induce IL-1β release in bone marrow derived macrophages (BMDM)
	Target	Not applicable
	Primary measurement	IL-1β level in cell culture supernatant quantified by ELISA
	Key reagents	Mouse IL-1β ELISA kit (Thermo; 88-7013-77)
	Assay protocol	See Methods
Library	Nature of the library	1,280 bioactives
	Size of the library	1,280 compounds arrayed in 96-well format as single compounds at 10mM in DMSO
	Source	Sigma LOPAC library (Sigma; LO4200)
	Concentration tested	40mM, 0.4% v/v DMSO
Screen	Format	96-well plates
	Plate controls	Negative control = DMSO
	Reagent/compound dispensing system	BMDMs were dispensed using P300 multichannel pipette (Eppendorf) at 100μL volume for cell seeding, then 20μL of media containing concentrated LPS were dispensed into each well for priming to achieve 200ng/mL LPS. 0.6μL of compounds were dispensed into 150μL of OPTI-MEM using P10 multichannel pipette (Eppendorf) and 50μL of compounds in OPTI-MEM were transferred to cell culture plate using P300 multichannel pipette.
	Detection instrument and software	Absorbance reading at 540nm and 450nm using iMARK microplate reader (Bio-Rad).
	Assay validation/QC	IL-1β release below background level for DMSO negative control
	Correction factors	Uncorrected (single replicate)
	Normalization	Normalized to in-plate nigericin (10μM)-induced IL-1β level (set at 2ng/mL) as the screen was performed in 3 different batches of BMDM
Post-HTS analysis	Hit criteria	IL-1β release greater than 0.2ng/mL (10% of nigericin)
	Hit rate	6.3% (80/1280)
	Retesting of initial actives	Original samples were rearrayed and retested at 40μM, 10μM, 2.5μM, and 0.625μM concentration using the primary screening assay; compounds with IL-1β release level >10% of nigericin at 40μM concentration upon retesting were labeled 'validated activators' (69 compounds); of these, compounds that maintained IL-1β release at 10μM, 2.5μM, and 0.625μM concentration were retested in dose-response mode (1 compound)
	Confirmation of hit purity and structure	Validated hit was repurchased and retested (Suppl. Fig. 3a)
Screen results	List of all screening positives	List of IL-1β release level at 40μM concentration (Supplementary Data 2; PrimaryScreens)
	List of validated compounds	List of IL-1β release level at 4 concentrations (Supplementary Data 2; ActivatorValidation)

Supplementary Table 3. Primary screen for suppressors of niclosamide- and nigericin-induced IL-1β release in THP-1 macrophages.

Category	Parameter	Description
Assay	Type of assay	Unbiased phenotypic screen for small molecules that suppress niclosamide- or nigericin-induced IL-1β release in THP-1 macrophage
	Target	Not applicable
	Primary measurement	IL-1β level in cell culture supernatant quantified by ELISA
	Key reagents	Human IL-1β ELISA kit (Thermo; 88-7261-77), Calcein, AM (Thermo; C3100MP)
	Assay protocol	See Methods
Library	Nature of the library	1,280 bioactives
	Size of the library	1,280 compounds arrayed in 96-well format as single compounds at 10mM in DMSO
	Source	Sigma LOPAC library (Sigma; LO4200)
	Concentration tested	40mM, 0.4% v/v DMSO
Screen	Format	96-well plates
	Plate controls	Negative control = DMSO
	Reagent/compound dispensing system	THP-1 cells were dispensed using P300 multichannel pipette (Eppendorf) at 100μL volume. 0.6μL of compounds were dispensed into 150μL of OPTI-MEM using P10 multichannel pipette (Eppendorf) and 50μL of compounds in OPTI-MEM were transferred to cell culture plate using P300 multichannel pipette. Concentrated activator (niclosamide or nigericin) in OPTI-MEM were transferred to cell culture plate at 5μL volume using P10 multichannel pipette to achieve a final concentration of 5μM niclosamide or 20μM nigericin.
	Detection instrument and software	Absorbance reading at 540nm and 450nm using iMARK microplate reader (Bio-Rad).
	Assay validation/QC	IL-1β release below background level for DMSO negative control
	Correction factors	Uncorrected (single replicate)
	Normalization	IL-1β release level of each compound was normalized to DMSO control for each plate and reported as % of DMSO control (no suppressor)
Post-HTS analysis	Hit criteria	Top 80 compounds based on % suppression of IL-1β release for each activator (niclosamide or nigericin), absent in activator activity (not part of the primary hit in activator screen; top 132)
	Hit rate	6.3% (80/1280)
	Retesting of initial actives	Original samples were rearrayed and retested at 40μM, 10μM, 2.5μM, and 0.625μM concentration for niclosamide- or nigericin-induced IL-1β release; compounds were also tested for toxicity using calcein cell viability assay at 40μM, 10μM, 2.5μM, and 0.625μM concentration; compounds that achieved >50% suppression of niclosamide-induced IL-1β release at 40μM, 10μM, 2.5μM, and 0.625μM concentration that were also >90% viable at all 4 concentrations were tested for suppression of AIM2 inflammasome activator poly(dA:dT); compounds that were biased for niclosamide over poly(dA:dT) (>25% difference) were filtered for <60% suppression of nigericin-induced IL-1β release at 40μM and tested in dose-response mode.
	Confirmation of hit purity and structure	Validated hits were repurchased and retested (Fig. 2b, c)
Screen results	List of all screening positives	List of % IL-1β release level at 40μM concentration relative to DMSO control (Supplementary Data 1; PrimaryScreens)
	List of validated compounds	List of % IL-1β release level at 4 concentrations relative to DMSO control (Supplementary Data 1; SuppressorValidationNiclosamide, SuppressorValidationNigericin)

Supplementary Table 4. Primary screen for suppressors of niclosamide- and nigericin-induced IL-1β release in BMDM.

Category	Parameter	Description
Assay	Type of assay	Unbiased phenotypic screen for small molecules that suppress niclosamide- or nigericin-induced IL-1β release in bone marrow derived macrophages (BMDM)
	Target	Not applicable
	Primary measurement	IL-1β level in cell culture supernatant quantified by ELISA
	Key reagents	Mouse IL-1β ELISA kit (Thermo; 88-7013-77)
	Assay protocol	See Methods
Library	Nature of the library	1,280 bioactives
	Size of the library	1,280 compounds arrayed in 96-well format as single compounds at 10mM in DMSO
	Source	Sigma LOPAC library (Sigma; LO4200)
	Concentration tested	40mM, 0.4% v/v DMSO
Screen	Format	96-well plates
	Plate controls	Negative control = DMSO
	Reagent/compound dispensing system	BMDMs were dispensed using P300 multichannel pipette (Eppendorf) at 100μL volume for cell seeding, then 20μL of media containing concentrated LPS were dispensed into each well for priming to achieve 200ng/mL LPS. 0.6μL of compounds were dispensed into 150μL of OPTI-MEM using P10 multichannel pipette (Eppendorf) and 50μL of compounds in OPTI-MEM were transferred to cell culture plate using P300 multichannel pipette. Concentrated activator (niclosamide or nigericin) in OPTI-MEM were transferred to cell culture plate at 5μL volume using P10 multichannel pipette to achieve a final concentration of 5μM niclosamide or 10μM nigericin.
	Detection instrument and software	Absorbance reading at 540nm and 450nm using iMARK microplate reader (Bio-Rad).
	Assay validation/QC	IL-1β release below background level for DMSO negative control
	Correction factors	Uncorrected (single replicate)
	Normalization	IL-1β release level of each compound was normalized to DMSO control for each plate and reported as % of DMSO control (no suppressor)
Post-HTS analysis	Hit criteria	Top 36 compounds with >80% suppression of niclosamide-induced IL-1β release and <20% suppression of nigericin-induced IL-1β release, absent in activator activity (IL-1β release < 0.2ng/mL)
	Hit rate	2.8% (36/1280)
	Retesting of initial actives	Original samples were rearrayed and retested at 40μM, 10μM, 2.5μM, and 0.625μM concentration for niclosamide- or nigericin-induced IL-1β release; compounds with >60% suppression of niclosamide-induced IL-1β release and <30% suppression of nigericin-induced IL-1β release at 10μM and 2.5μM were selected
	Confirmation of hit purity and structure	Validated hits were repurchased and retested (Suppl. Fig. 7a,b)
Screen results	List of all screening positives	List of % IL-1β release level at 40μM concentration relative to DMSO control (Supplementary Data 2; PrimaryScreens)
	List of validated compounds	List of % IL-1β release level at 4 concentrations relative to DMSO control (Supplementary Data 2; SuppressorValidationNiclosamide, SuppressorValidationNigericin, SuppressorValidationBiased)

Supplementary Table 5. Oligonucleotide pairs used to generate gene-specific single guide RNA (sgRNA) in the LentiCRISPR_v2 vector.

Target Sequence	Oligo 1	Oligo 2
N.Ctl1	CACCGACGGAGGCTAAGCGTCGCAA	AAACTTGCGACGCTTAGCCTCCGTC
N.Ctl2	CACCGCGCTTCCGCGGCCCGTTCAA	AAACTTGAACGGGCCGCGGAAGCGC
NLRP3	CACCGGTCTGATTCCGAAGTCACCG	AAACCGGTGACTTCGGAATCAGACC
ASC	CACCGCATGTCGCGCAGCACGTTAG	AAACCTAACGTGCTGCGCGACATGC
CASP1	CACCGCTAAACAGACAAGGTCCTGA	AAACTCAGGACCTTGTCTGTTTAGC
